# Advanced Development of Primary Pancreatic Organoid Tumor Models for High-Throughput Phenotypic Drug Screening

**DOI:** 10.1177/2472555218766842

**Published:** 2018-04-19

**Authors:** Shurong Hou, Hervé Tiriac, Banu Priya Sridharan, Louis Scampavia, Franck Madoux, Jan Seldin, Glauco R. Souza, Donald Watson, David Tuveson, Timothy P. Spicer

**Affiliations:** 1 The Scripps Research Institute Molecular Screening Center, Department of Molecular Medicine, Scripps Florida, Jupiter, FL, USA; 2 Cancer Center, Cold Spring Harbor Laboratory, Cold Spring Harbor, NY, USA; 3 Greiner Bio-One North America, Inc., Monroe, NC, USA; 4 Nano3D Biosciences, Inc. and University of Texas Health Science Center at Houston, Houston, TX, USA; 5 Dana-Farber Cancer Institute, Boston, MA, USA; * These authors contributed equally to this work; † Amgen, Inc., Thousand Oaks, CA, USA; ‡ Co-communicated by D.T. and T.P.S.

**Keywords:** organoid, pancreatic, cancer, phenotypic, HTS

## Abstract

Traditional high-throughput drug screening in oncology routinely relies on two-dimensional (2D) cell models, which inadequately recapitulate the physiologic context of cancer. Three-dimensional (3D) cell models are thought to better mimic the complexity of in vivo tumors. Numerous methods to culture 3D organoids have been described, but most are nonhomogeneous and expensive, and hence impractical for high-throughput screening (HTS) purposes. Here we describe an HTS-compatible method that enables the consistent production of organoids in standard flat-bottom 384- and 1536-well plates by combining the use of a cell-repellent surface with a bioprinting technology incorporating magnetic force. We validated this homogeneous process by evaluating the effects of well-characterized anticancer agents against four patient-derived pancreatic cancer KRAS mutant-associated primary cells, including cancer-associated fibroblasts. This technology was tested for its compatibility with HTS automation by completing a cytotoxicity pilot screen of ~3300 approved drugs. To highlight the benefits of the 3D format, we performed this pilot screen in parallel in both the 2D and 3D assays. These data indicate that this technique can be readily applied to support large-scale drug screening relying on clinically relevant, ex vivo 3D tumor models directly harvested from patients, an important milestone toward personalized medicine.

## Introduction

Establishing better and more predictive cancer models to evaluate current and future chemotherapeutics is of high priority. Creating three-dimensional (3D) culture systems utilizing patient-derived tumors for rapid testing in high-density format would constitute an important achievement toward precision medicine and regenerative therapies. Traditional monolayer cancer models exhibit an unrestrained proliferation phenotype, which has proven limited value in predicting clinical response to novel agents.^1^ 3D ex vivo tumor models are able to better recapitulate the features of in vivo cancer, such as cell–cell interactions, cell–matrix interactions, hypoxia, heterogeneity of tumor, drug penetration, and drug resistance.^2–6^ Therefore, physiologically relevant 3D cell culture has been recognized as a potential bridge between traditional in vitro two-dimensional (2D) culture and in vivo animal studies.^7,8^ It is thus critical to advance the development of scalable and affordable methods of producing 3D spheroids and/or organoids suitable for high-throughput cancer drug discovery.

Patient tumor-derived organoid models can be readily isolated and could be a formidable model for precision medicine testing with an appropriate 3D high-throughput screening (HTS) strategy. Therefore, we set out to determine the conditions for 3D screening of cancer cells and associated fibroblasts. By definition, spheroids are usually self-assembling or are forced to grow as cell aggregates starting from single cell suspensions of established cell lines.^4^ Various methods for 3D cancer cell culture have been developed, including spontaneous cell aggregation, hanging drop, spinner culture, pellet culture, cultures using cell-repellent plates and/or external force, and scaffold-based cultures.^9–11^ Organoids, on the other hand, are defined as 3D cellular clusters typically derived specifically from primary tissue, embryonic stem cells, or induced pluripotent stem cells.^2,3,12^ Organoid cultures traditionally rely on artificial extracellular matrices (ECMs) such as Matrigel to facilitate their self-organization into structures that closely mimic the features of in vivo tissue.^10,13^

Due to the difficulty in early diagnosis and lack of effective treatment, pancreatic cancer remains one of the most common causes of cancer-related death, with an overall 5-year survival rate of less than 7%.^14–16^ Genetic alterations in oncogenic KRAS and tumor suppressors TP53, CDKN2A, SMAD4, ARID1A, and MLL3 are found in pancreatic cancers.^7^ Cancer-associated fibroblasts (CAFs), as part of the tumor microenvironment, also play an important role in tumor initiation and progression, as well as drug efficacy.^17–19^ As such, in this study, we focused our effort on culturing two pancreatic cancer cells and two CAFs. All were established directly from pancreatic cancer patient tissue^13^ and are furthermore referred to as the following: hT1 from resected primary tumor and genotyped as Kras^G12V^, P53^loss^, SMAD4^loss^, and CDKN2A^hom del^; hM1 from a resected metastatic lung lesion containing KRAS^G12D^ and P53^R175H^; and hT1-CAF and hM1-CAF, which are sv40-immortalized KRAS wild type with no genetic mutations in KRAS or tumor suppressor genes and are fibroblast lines derived from the same tumor mass as hT1 and hM1 cells.

The essential requirement for implementation of 3D tumor models for HTS therapeutic screening is to efficiently and economically produce and/or seed uniform spheroids or organoids in high-density microplates and to achieve acceptable HTS assay performance.^4^ In this study, we focus on the development of HTS-amenable 3D pancreatic cancer cell culture in standard flat-bottom well plates by combining a cell-repellant surface with a bioprinting technology that relies on magnetic force.^13,20^ We applied a parallel 384-well HTS approach to test the National Cancer Institute (NCI)-approved oncology set and separately ~3300 approved drugs versus 4 pancreatic cancer-related cell models (hT1, hT1-CAF, hM1, and hM1-CAF) in both 2D and 3D formats. These tests and outcomes will be described and ultimately provide the basis for future studies aimed at the development of effective medication for pancreatic cancer.

## Material and Methods

### Cells

Human colorectal adenocarcinoma cell line HT-29 (ATCC no. HTB-38) and human pancreatic epithelial carcinoma cell line PANC-1 (ATCC no. CRL-1469) were purchased from ATCC and cultured according to the manufacturer’s suggested protocol. Primary human pancreatic ductal cells hM1 and hT1, and cancer-associated fibroblasts hM1-CAF and hT1-CAF (immortalized by SV40), were generated from tissues of pancreatic cancer patients in the laboratory of Dr. Tuveson.^13^ These cells were cultured in flasks and expanded as 2D monolayers in RPMI 1640 (part no. 10-040-CV, Life Technology, Carlsbad, CA) with 10% serum (part no. 97068-085, VWR, Radnor, PA) and 1× Anti-Anti (part no. 15240-062, Life Technology) at 37 °C, 5% CO_2_, and 95% relative humidity. They were harvested and utilized in this format for the purpose of both 2D and 3D testing, using the same media as described above.

### Compound Library

A collection of almost 3300 clinically approved drugs obtained from multiple vendors were assembled at the Scripps Research Institute Molecular Screening Center (SRIMSC) and reformatted into 1536-well source plates for automated robotic screening. In addition, the NCI-approved oncology drug set of 114 compounds was obtained directly from the NCI and included. Note that each of these compounds has been approved either by the FDA, the European Medicines Agency, or the Japanese Pharmaceuticals and Medical Devices Agency.^21^

### 2D Cell Viability Assay

A 1536-well 2D cell viability assay was optimized and implemented, which determines the number of viable cells based on the amount of ATP present using commercially available luminescence detection reagent CellTiter-Glo (part no. G7573, Promega, Madison, WI) as previously described.^22^

Prior to plating, cells were grown to 80% confluence in RPMI 1640 complete growth media. After washing once with phosphate-buffered saline (PBS), cells were detached by TrypLE (part no. 12604021, Life Technologies) and centrifuged at 300*g* for 5 min. Cells were suspended and filtered through cell strainer. Two hundred cells in 5 µL of culture media were seeded in 1536-well plates (part no. 789173-F, Greiner Bio-One, Monroe, NC). After incubation of the assay plates overnight (~14 h), cells were treated with compounds and vehicle (10 nL, 0.02% DMSO). Cell viability was assessed after 72 h of incubation using CellTiter-Glo reagent according to manufacturer’s instructions. The ViewLux microplate reader (PerkinElmer, Waltham, MA) was used to quantitate luminescence signal. IC_50_ values of five pharmacological control compounds (doxorubicin, gemcitabine, SN-38, 5-fluorouracil, and oxaliplatin, all purchased from Sigma, St. Louis, MO) were determined by fitting the concentration–response curve (CRC) data with a four-parameter variable-slope method in GraphPad Prism (GraphPad Software, San Diego, CA).

### 3D Culture and 3D Viability Assay

The 3D cell viability assay was initially developed and screened in a 384-well format and then further miniaturized into a 1536-well format. The 3D viability assay uses a detection reagent more adapted to spheroids that features a tailored lysis buffer (CellTiter-Glo 3D, part no. G9683, Promega). Both assays were optimized by testing different variables, including cell number, printing time, incubation time, time of drug addition, and NanoShuttle amount. NanoShuttle, a reagent obtained from Nano3D Biosciences (Houston, TX), contains gold and iron oxide-laden nanoparticles attached to poly-l-lysine, which nonspecifically attached to the cell membrane of all eukaryotic cells. A detailed stepwise protocol of the final conditions is presented in **Table 1**. Cells were grown to 80% confluence in RPMI 1640 complete growth media and labeled with NanoShuttle-PL (part no. 657846, Greiner Bio-One) overnight (~16 h) in the T175 flasks. The second day, labeled cells were harvested and filtered through a 70 µm cell strainer. For all types of cells, 2500 cells in 25 µL culture media were seeded in 384-well Greiner Bio-One flat-bottom, cell-repellent plates (specialized version of part no. 781976, Greiner Bio-One), and 1250 cells in 5 µL culture media were seeded in 1536-well Greiner Bio-One flat-bottom, cell-repellent plates (part no. 789979, Greiner Bio-One). After putting the assay plate on top of the magnetic drive for 4 h, followed by incubation for 24 h to allow cells to form 3D structures, cells were treated with compounds or vehicle (50 or 10 nL, 0.1% DMSO). Cell viability was assessed after 72 h of incubation using CellTiter-Glo 3D reagent according to the manufacturer’s instructions. CRC and IC_50_ values of 5 pharmacological control compounds were used as the guide for assay optimization and drug screening. As a point of comparison, we also tested these cells using Corning spheroid plate technology (part no. 3830, Corning Inc., Corning, NY).^4^ The Corning spheroid-based assay follows the same protocol except that (1) cells are not labeled with NanoShuttle and also do not need a magnetic drive, and (2) a brief centrifugation was performed immediately after seeding cells into the Corning spheroid plates to facilitate spheroid formation.

**Table 1.** Stepwise Protocol for the 2D and n3D Bioprinting-Based Assay.

Step	3D Assay	2D Assay	Comments
1	Add 0.6 mL of NanoShuttle to cells in each T175 flask when cells reach 80% confluency.	Prior to plating, grow cells to 80% confluence in complete growth media.	
2	Incubate overnight at 37 °C, 5% CO_2_, 95% relative humidity.	NA	
3	Harvest and seed cells into ULA plates (384-well plate: 25 µL, 2500 cells per well; 1536-well plate: 5 µL, 1250 cells per well).	Harvest and seed cells into 1536-well TC-treated plates (5 µL, 200 cells per well).	BioRAPTR FRD (Beckman Coulter, Brea, CA) for cell dispense
4	Put the plates atop of the 384- or 1536-well magnetic drive for 4 h and incubate them at 37 °C, 5% CO_2_, 95% relative humidity.	NA	
5	Incubate for 24 h at 37 °C, 5% CO_2_, 95% relative humidity.	Incubate overnight (16 h) at 37 °C, 5% CO_2_, 95% relative humidity.	
6	Add controls and test compounds (50 nL per well for 384-well plate or 10 nL per well for 1536-well plate).	Add controls and test compounds (10 nL per well).	PinTool (GNF) for compound transfer; 2 µM final screening concentration (0.15% DMSO)
7	Incubate for 72 h at 37 °C, 5% CO_2_, 95% relative humidity.	Incubate for 72 h at 37 °C, 5% CO_2_, 95% relative humidity.	Plates kept in a humidified chamber
8	Add CellTiter-Glo 3D detection reagent (25 µL per well for 384-well plate or 5 µL per well for 1536-well plate).	Add CellTiter-Glo detection reagent (5 µL per well).	3D CellTiter-Glo for 3D assay, normal CellTiter-Glo for 2D assay
9	Centrifuge plates for 5 min and incubate for 60 min at room temperature.	Centrifuge plates for 2 min and incubate for 10 min at room temperature.	
10	Read luminescence.	Read luminescence.	On ViewLux (PerkinElmer)

NA, not applicable; TC, tissue culture.

The formation of the 3D structure was confirmed by Z-stack analysis using an IN Cell Analyzer 6000 confocal high-content reader, using cells stained with Hoechst or CellTracker Green in a Greiner Bio-One flat-bottom, cell-repellent plate designed for imaging. Multiple Z-stack images were taken at 5 or 2 µm increments and aligned in ImageJ to generate an intensity projection biased by color scale (**Suppl. Fig. S1**).

### HTS Campaign and Data Processing

A set of ~3300 approved drugs and the NCI-approved oncology set were screened in 384-well plate format in 3D or in 1536-well plate format in 2D, at 2 µM nominal concentration against four pancreatic cancer-related cell models (hM1, hM1-CAF, hT1, and hT1-CAF). All data files obtained were uploaded into Scripps’ institutional database for individual plate quality control and hit selection. Assay plates were determined acceptable only if Z′ > 0.5.^5^ Compound activity was normalized on a per-plate basis using the following equation:^23^

$$\%inhibition=100\times (1-\frac{Test\;Well-Median\;High\;Control}{Median\;Low\;Control-Median\;High\;Control})$$

*Test Well* refers to those wells with cells treated with test compounds. *High Control* is defined as wells containing medium only (100% inhibition), and *Low Control* wells contain cells treated with DMSO only (0% inhibition).

High and low controls were applied for assay quality evaluation in terms of Z′.^5^ Day-to-day assay response and stability was assessed using five pharmacological control compounds that we tested for CRC and required to be within threefold of the expected IC_50_, on an experimental basis, for each cell model. An interval-based hit cutoff was used to define active compounds for each assay. This cutoff is calculated as the average percent inhibition plus three times the standard deviation (SD), of all the tested compounds except those showing percent inhibition higher than the average + 3SD of the high controls or percent inhibition lower than the average – 3SD of the low controls.^24,25^ A four-way Venn diagram was used to analyze the hit compounds in hM1, hM1-CAF, hT1, and hT1-CAF assays. The tool is freely available at http://www.pangloss.com/seidel/Protocols/venn4.cgi. Active hits against each of the assays were chosen to determine their IC_50_.

The selected drugs were prepared as 10-point, threefold serial dilutions and tested against four pancreatic cancer-derived cells (hM1, hM1-CAF, hT1, and hT1-CAF) in 2D or 3D format in triplicate starting from 5 µM nominal concentration. For each test compound, percent inhibition was plotted against compound concentration. A four-parameter equation describing a sigmoidal dose–response curve was then fitted with adjustable baseline using Assay Explorer software (Symyx Technologies, Santa Clara, CA). The reported IC_50_ values were generated from fitted curves by solving for the *x* intercept value at the 50% inhibition level of the *y* intercept value. In cases where the highest concentration tested (i.e., 5 µM) did not result in greater than 50% cytotoxicity, the IC_50_ was deemed as greater than 5 µM. The heat map of the activity of 114 NCI oncology drugs against each of the patient-derived cultures in both 2D and 3D format was plotted using Tibco Spotfire software (TIBCO Software, Palo Alto, CA).

## Results and Discussion

### Forming 3D Structures

**Figure 1.**
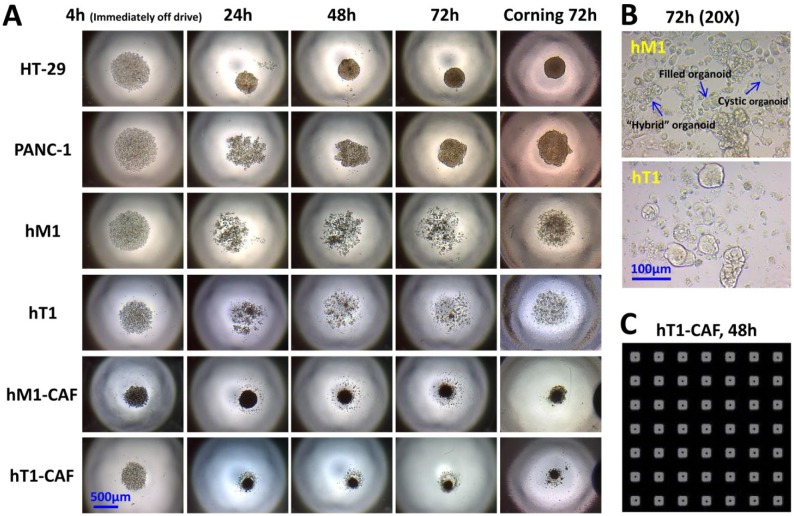
A panel of pancreatic cancer-derived cells was evaluated for their ability to form 3D structures using n3D bioprinting technology. (**A**) The 3D structure formation of primary pancreatic cancer cells (hT1 and hM1), their associated fibroblasts (hT1-CAF and hM1-CAF), and standard cell lines (HT-29 and PANC-1) was monitored using standard microscopy (4× objective) in a Greiner Bio-One 384-well cell-repellent, flat-bottom plate. These cells were also cultured in 384 Corning U-bottom spheroid plates as a point of comparison. (**B**) Enlarged images of hM1 and hT1 3D culture using a 20× objective representing small organoid-like structures in primary pancreatic cancer culture. (**C**) A portion of the full 384-well plate image obtained using the Scripps HIAPI instrument is shown to demonstrate that homogeneous hT1-CAF spheroids were formed in each well of a 384-well plate using bioprinting technology.

**Figure 2.**
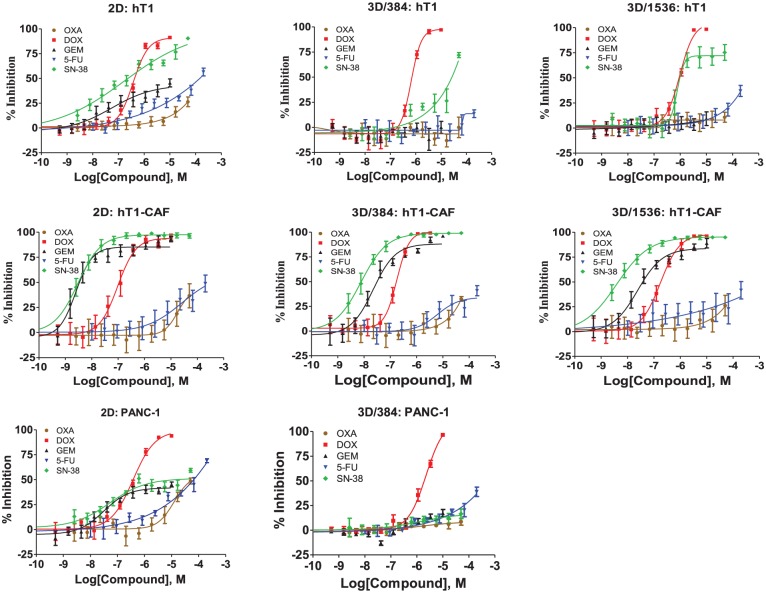
CRCs for five control compounds (oxaliplatin, doxorubicin, gemcitabine, 5-fluorouracil, and SN-38) versus hT1, hT1-CAF, and PANC-1 in 2D and 3D formats (384 and 1536 wells). Each curve represents the mean and standard deviation of four replicates in 384 wells or 16 replicates in 1536 wells.

Pancreatic cancer is recognized as a heterogeneous cancer with genotypic and phenotypic diversity observed not only between patients but also within a tumor.^7,11^ In this study, we employed the bioprinting technique using n3D technology and grew spheroids in flat-bottom, cell-repellent plates in the absence of exogenous ECM components. For this effort, a panel of pancreatic cancer-derived cells was evaluated for their ability to form spheroids or organoids. We also included the well-characterized human pancreatic epithelial carcinoma cell line, PANC-1, as a point of reference to the hT1 and hM1 as representatives of primary and metastatic pancreatic cancer cells, respectively, along with their corresponding CAFs. In addition, the human colorectal adenocarcinoma cell line HT-29 was used as a control cell line due to its inclination to form spheroids.^26^ Some cells are indeed more inclined to form spheroids than others (**Fig. 1**), which is easily observed by bright field microscopy. As anticipated, HT-29 cells formed extremely compact spheroids with a well-defined surface, while PANC-1 cells formed relatively compact and round spheroids, which are a bit more amorphous.^6^ The two CAF lines readily formed into compact spheroids in each well, whereas their corresponding pancreatic cancer primary cells (hT1 and hM1) presented only as loose cell clusters, which were confirmed to be organoid-like 3D structures in higher-magnification images. The different 3D-forming ability among the tested panel of cells is likely due to the different expression levels of adhesion molecules, such as β1-integrin and E-cadherin, and the interaction of β1-integrin with ECM proteins, which are required during spheroid formation, similarly to what has been demonstrated in hepatoma, PANC-1, and breast cancer spheroid formation.^6,9,27,28^ In particular, the fibronectin–integrin interaction probably plays an important role in the formation of tight fibroblast spheroids, because fibroblasts are known to produce many components of the interstitial ECM, such as fibronectin, in order to maintain the integrity of connective tissue. Although hT1 and hM1 did not form obvious spheroids as did the other cells, a closer morphological evaluation revealed the presence of small organoid-like 3D structures in their cultures, as marked in **Figure 1B**. In this culture system, primary pancreatic cancer cells hT1 and hM1 formed several types of organoids, including cystic, filled, and hybrid organoids, that are similar to the ones reported by others in organoid models using the Matrigel culture system,^13^ thus proving that we generated bona fide organoids in the absence of ECMs using the n3D bioprinting technology. Additionally, while this study focused on the use of cell-repellent surfaces for 3D technologies, reported outcomes when testing hM1 cells in either Matrigel or n3D formats are available.^29^ The same panel of cells cultured in Corning spheroid plates preserved the morphology found using n3D bioprinting technology (**Fig. 1A**), which confirms that the difference in spheroid formation among the panel of tumor-derived cells is not associated with the plate type or n3D reagent, but rather related to their different cell properties. In addition, the same morphology of spheroids or organoids was observed when adapting those models into a 1536-well plate (**Suppl. Fig. S1**), which also conveyed virtually identical and overlapping sensitivity profiles compared with the 384-well formats (**Fig. 2**).

### 2D and 3D Assay Optimization

Monolayer assays using four pancreatic patient-related cells (hT1, hT1-CAF, hM1, and hM1-CAF) were directly implemented in the 1536-well format and optimized for cell seeding density, incubation time, and DMSO tolerance (**Table 1**). Each 2D assay requires the seeding of 200 cells in 5 µL of media within each well of 1536-well tissue culture (TC)-treated plates, which were further incubated overnight (~14 h) to let cells attach prior to adding 10 nL of test compounds. Plates were then incubated for another 72 h before adding CellTiter-Glo reagent to evaluate the cell viability.

The 3D assays were first developed in 384-well plate format and optimized by testing different variables, including printing, incubation, and drug addition time, as well as amounts of NanoShuttle (data not shown). The adequate cell density was evaluated in order to ensure that cell proliferation remained in the linear phase of growth throughout the entire assay period. Twenty-five hundred cells per well were chosen for each type of cell. Neither the spheroid morphology nor the response to pharmacologic controls changed when the printing time was decreased from 24 h to 4 h, or while the NanoShuttle amount decreased down to 50% of the suggested amount (600 µL per T175 flask). Similar to our former spheroid-based assay, the incubation time before compound addition in this n3D-formatted assay was initially set to be 48 h.^4^ We were able to shorten this incubation time to 24 h without affecting the spheroid morphology and the response of pharmacologic controls.

The 1536-well assays adopted exactly the same protocol described above, adjusting cell number and volumes to this miniaturized format, as shown in **Table 1**. Both 2D and 3D assays eventually last 4 days from cell seeding to the end point. To eliminate the edge effect, we implemented a supersaturated humidified condition using our GNF system incubators.

The homogeneity of spheroids across the whole plate has been illustrated using hT1-CAF spheroids as an example, as shown in **Figure 1C**. These images were obtained using the Scripps Plate Auditor.^30^ Forming homogenous spheroids in size and shape across an entire screening plate is necessary to obtain the robust assay statistics required for drug testing. The effects of five pharmacological controls were evaluated side by side in 2D and 3D assays. The matched pair hT1 and hT1-CAF shown in **Figure 2** constitute representative examples of pancreatic cancer cells and cancer-associated fibroblasts. Compared with their respective 2D tests, both PANC-1 and hT1 in 3D format presented significantly different responses to the five control drugs, generally demonstrating less efficacy. The 3D models of pancreatic cancer cells showed the expected drug resistance observed in other studies.^6,31,32^ In contrast, the hT1-CAFs behaved similarly in both 2D and 3D formats. The same trend was also observed in hM1 and hM1-CAF cells. For example, gemcitabine, a first-line treatment for pancreatic cancer, showed ~400-fold less sensitivity in 3D hM1 models than the corresponding 2D hM1 culture, but only ~5-fold less sensitivity in the 3D hM1-CAF spheroids than 2D hM1-CAF. Moreover, similar cytotoxicity profiles were obtained for the five control drugs in both 384-well and1536-well 3D formats, suggesting that the miniaturization did not impact the ability of the assay to profile compounds. To further validate this 3D bioprinting culture technology, the dose response of five control drugs was tested side by side in PANC-1, hT1, and hT1-CAF 3D cultures generated with n3D bioprinting technology and the Corning spheroid round-bottom plate technology. As shown in **Supplemental Figure S2**, virtually identical responses were produced using these two different 3D culture technologies, providing further evidence that either technology is appropriate for high-throughput drug screening.

### Testing the NCI Oncology Drug Set

To further validate the 3D culture-based HTS assay, 114 NCI oncology drugs were tested in 384-well plates, against each of the aforementioned pancreatic cancer-associated cells in triplicate, as 10-point, threefold serial dilutions starting from a 5 µM nominal concentration. This same procedure was also done in 1536-well plates for 2D culture.

For 3D, average Z′ values of 0.82, 0.77, 0.86, and 0.85 were obtained for hT1, hT1-CAF, hM1, and hM1-CAF, respectively. We identified 7, 16, 5, and 14 compounds with IC_50_ < 1 µM against hT1, hT1-CAF, hM1, and hM1-CAF, respectively; 4 of these (romidepsin, bortezomib, carfilzomib, and homoharringtonine) displayed an IC_50_ < 1 µM against all four 3D models. For 2D, average Z′ values of 0.80, 0.69, 0.84, and 0.66 were obtained for hT1, hT1-CAF, hM1, and hM1-CAF, respectively. We identified 9, 27, 33, and 32 compounds with IC_50_ < 1 µM against hT1, hT1-CAF, hM1, and hM1-CAF, respectively. The selectivity of those active compounds (identified as IC_50_ < 1 µM) on 2D and 3D models can be visualized using a four-way Venn diagram (**Suppl. Fig. S3A**). Fifty-six compounds are noncytotoxic to all four 3D models meaning the maximum percent inhibition was less than the calculated hit cutoff obtained from the average plus 3SD of DMSO wells, which was 32.95% for the hT1 assay, 23.30% for the hT1-CAF assay, 10.95% for the hM1 assay, and 14.10% for the hM1-CAF assay.

**Figure 3.**
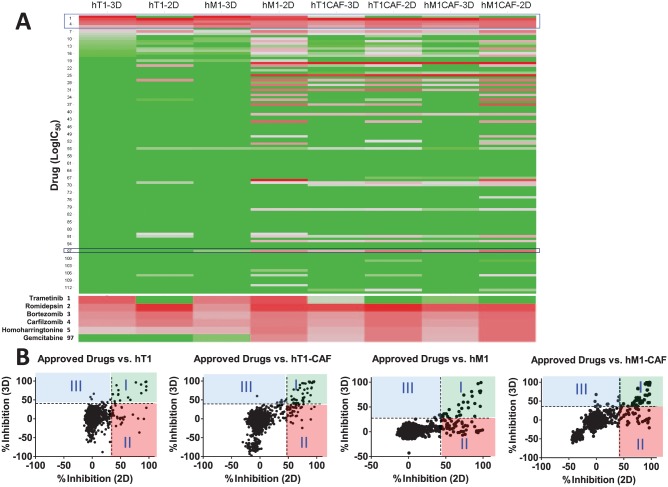
(**A**) Heat map of the activity of 114 NCI oncology drugs associated with each of the four pancreatic cancer-associated cells in 3D and 2D formats assessed by corresponding log IC_50_ values (red = increased potency; green = decreased potency). The responses to the most potent drugs, trametinib, romidepsin, bortezomib, carfilzomib, and homoharringtonine, plus gemcitabine, the first-line drug for treating pancreatic cancer, are highlighted below the graph. (**B**) The correlation plot of the percent inhibition values of the approved drug library tested at 2 µM in the 3D and 2D models of each pancreatic cancer-associated cell.

**Figure 4.**
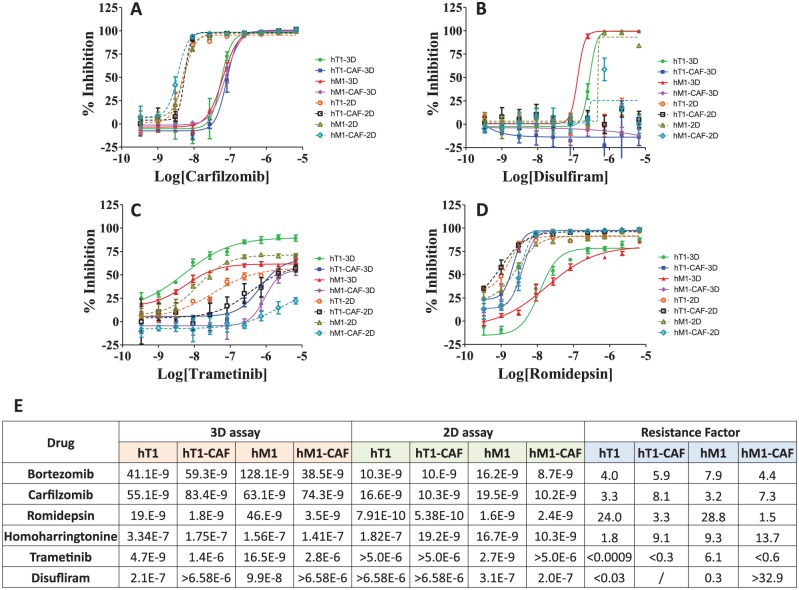
CRCs of (**A**) carfilzomib, (**B**) disulfiram, (**C**) trametinib, and (**D**) romidepsin tested in the 3D and 2D models of each of the four pancreatic cancer-associated cells. The curve represents the mean and standard deviation in triplicate. IC_50_ values of those drugs, including other potent inhibitors in each cell model, and the corresponding resistance factor for each cell type are summarized in **E**.

To easily view each drug’s selectivity and sensitivity in 2D and 3D models of the four cultures, the activity of the 114 oncology drugs against each of the models was plotted in a heat map using log IC_50_ as the measurement (**Fig. 3A**). Similar to the 2D result, some drugs showed strong cytotoxicity in all four 3D models, while others demonstrated certain cell-specific selectivity. In 2D or 3D assays, the two CAF lines, that is, hT1-CAF and hM1-CAF, exhibited similar drug sensitivity. In contrast, the two primary cancer cells, hT1 and hM1, are more resistant to cytotoxic drugs than corresponding CAF lines. As anticipated, the 3D culture models are more resistant to these oncology drugs than the corresponding 2D models. Strikingly, microtubule modulators such as cabazitaxel, docetaxel, vinblastine, vincristine, paclitaxel, and ixabepilone are highly active (IC_50_ in the nanomolar range) in hM1-2D assay but show little to no activity in the hM1-3D assay. This may not be surprising considering that resistance to paclitaxel was observed in some solid tumors, and the protection effects against microtubule-directed chemotherapeutic agents have also been reported on breast cancer cells when introducing ECM proteins to the culture system.^33^ The significant loss of cytotoxicity by microtubule modulators in ECM-rich 3D cultures observed in this study is consistent with expectations, which further supports an ECM-mediated drug resistance mechanism in 3D cell culture.^33^ This phenomenon was also observed in hM1-CAF to some degree, but not in hT1 and hT1-CAF. Bortezomib, carfilzomib, romidepsin, and homoharringtonine present strong inhibitory activity (0.5–330 nM range) against all four patient-derived cultures in both 3D and 2D format (**Fig. 4**). Among those drugs, bortezomib and carfilzomib depicted a lower degree of 3D to 2D resistance in pancreatic cancer cells, while romidepsin showed the highest degree of resistance (24-fold) in pancreatic cancer 3D models. To assess the degree of resistance of the 3D model for a specific drug when compared with its 2D counterpart, we introduce the concept of “resistance factor,” which is simply defined as the ratio between the IC_50_ obtained for a given drug in a 3D and its corresponding 2D assay, respectively. For example, as for hT1 and hM1 cells, the resistance factor is 4.6 ± 2.2 for two proteasome inhibitors, bortezomib and carfilzomib, and 26.4 ± 3.4 for romidepsin. Looking closely at the correlation plot of the maximum percent inhibition of oncology drugs in 3D format and the corresponding 2D assay (**Suppl. Fig. S3B and Suppl. Table S1**), for each cell type, more compounds are 2D biased (hit only in 2D assay) and fewer compounds are 3D biased (hit only in 3D assay). Trametinib, approved for metastatic melanoma with known BRAF mutations, shows ~1000-fold more activity in hT1-3D assay than the corresponding hT1-2D assay. In comparison with another laboratory’s results, similar differential activity of trametinib was reported in PANC-1 2D and 3D culture models.^32^ It is also a cytotoxic compound that is more effective against pancreatic cancer cells over its counterpart CAFs in 3D assays (**Figs. 3A** **and** **4**). Taken together, the robust assay statistics generated and the expected bias toward lower toxicity effects observed in 3D assays indicate that our suite of parallel 3D HTS assays is adequate to conduct the pilot screen of a set of approved drugs.

### Approved Drug Screen

A total of 3290 approved drugs were screened in 384-well plate format at a 2 µM nominal concentration against all four types of cells in 3D culture models. Results are presented in **Supplemental Figure S4**. The average Z′ was 0.80, 0.75, 0.91, and 0.82 for hT1, hT1-CAF, hM1, and hM1-CAF, respectively. Similar Z′ values were achieved in 1536-well format when screening the library in 2D.

Due to the high compound activity observed during these screens, we applied an interval-based hit cutoff to identify hits.^24^ This allowed us to apply a reasonable cutoff parameter while alleviating the bias (i.e., negative activity scatter) observed below the low control, in particular seen in the CAF assays (**Suppl. Fig. S4A**). The hit cutoffs were 40.21% for hT1, 39.41% for hT1-CAF, 27.34% for hM1, and 35.69% for hM1-CAF. These identified 26 and 40 hits for hT1 and hM1, and 53 and 61 hits for hT1-CAF and hM1-CAF, respectively; that is, roughly 1%–2% of tested drugs were active against each 3D model. When comparing the 3D screen results with the 2D screening data (**Fig. 3B**), the majority of the compounds were inactive in both formats. We found that ~50% of hits from the 2D screen are also active in the corresponding 3D screen, but much more hits preferentially target 2D cells than 3D culture.

Overall, 14 compounds are active in all the four 3D assays and 89 compounds hit at least one 3D cell model (**Suppl. Fig. S4B**). Out of the 89 compounds (88 unique ones), 31 compounds overlapped with 114 NCI oncology drugs, which have already been tested in dose–response format. The remaining 57 drugs were cherry-picked and tested in dose response.

### Dose Response of Selected Hits

Fifty-four available compounds were prepared as 10-point, 3:1 serial dilutions and tested starting from ~5 µM nominal concentrations in the four aforementionned parallel 3D assays in triplicate. Consistent with previous efforts, satisfactory Z′ data were obtained, with an average of 0.78, 0.73, 0.90, and 0.85 in hT1, hT1-CAF, hM1, and hM1-CAF assays, respectively. We identified 5, 13, 3, and 17 compounds with an IC_50_ < 1 µM against the 3D format of hT1, hT1-CAF, hM1, and hM1-CAF, respectively. A Venn diagram analysis of those compounds with an IC_50_ < 1 µM (**Suppl. Fig. S4C**) revealed that two drugs, proscillaridin A and brilliant green, have IC_50_ < 1 µM against all four 3D cell models, seven drugs are only active in both 3D CAFs (hT1-CAF and hM1-CAF), and one drug is only active against both 3D pancreatic cancer cells (hT1 and hM1). There were 0, 2, 0, or 7 compounds that only hit hT1, hT1-CAF, hM1, and hM1-CAF, respectively.

Among the seven drugs that have an IC_50_ < 1 µM to both 3D CAFs, two belong to the statins family, cerivastatin and pitavastatin, which demonstrated a 7- to 34-fold sensitivity increase to 3D CAFs compared with the corresponding 3D cancer cells. Three of them are cardiac glycosides, quabain, digoxin, and lanatoside A, which presented only a three- to sixfold sensitivity enhancement to 3D CAFs compared with corresponding 3D cancer cells. The drug that only hit 3D pancreatic cancer cells and not CAFs is disulfiram, an FDA-approved drug indicated for alcoholism treatment. Disulfiram, an aldehyde dehydrogenase inhibitor, has also been shown to inhibit other actvities, such as in the proteasome, DNA topoisomerase, matrix metalloproteinase, and ABC drug transporter proteins.^34,35^ Several studies have reported its antitumor and chemotherapy-sensitizing activities in various cancer cells, including prostate cancer cells, triple-negative breast cancer cells, and NSC lung cancer cells.^34–39^ In this study, disulfiram displayed strong inhibitory activity against hT1-3D with an observed IC_50_ of 210 nM and no activity toward hT1-2D (**Fig. 4B**). It is also very active in both hM1-3D and hM1-2D assays (98 and 313 nM, respectively). It is less active against CAFs in both 3D and 2D formats. With the compendium of data favoring such, disulfiram has been prioritized in follow-up organoid and mouse model testing (data forthcoming).

In summary, we have developed and validated 3D cell culture using n3D bioprinting technology for HTS therapeutic screening to advance the discovery of clinically useful antipancreatic cancer drugs against solid tumor primary cells. Established 3D cell culture models reflecting the in vivo tumor and drug resistance may serve as more relevant screening models searching for effective chemotherapeutics. We identified multiple compounds from the pilot screen of the approved drug library, such as proteasome inhibitors bortezomib and carfilzomib, histone deacetylase inhibitor romidepsin, and protein synthesis inhibitor homoharringtonine, that demonstrate similar strong cytotoxic effects across all four pancreatic cancer patient-derived cells, and several drugs that are clearly cell line specific in this test scenario, including CAF-selective statins and cardiac glycosides, as well as pancreatic cancer cell-selective trametinib and disulfiram. As anticipated, most of the tested drugs were less active in 3D, but a few drugs showed preferential cytotoxicity against 3D models over 2D culture, which proved to be both cell and drug dependent. The preference of disulfiram to hit 3D models over 2D models and its addition to the clinical studies in metastatic pancreatic cancer,^40^ prostate cancer,^41^ and glioblastoma^42,43^ seem to confirm that we are using a more phenotypically relevant strategy that could translate into the development of precision medication initiatives for oncology research. The screening approach presented here demonstrated robustness as well as the ability to quickly identify and elucidate potential therapeutic drugs against pancreatic cancer. The different drugs identified here may be thought of as early leads, as they should be amenable for rapid translation to clinical studies because of their well-known pharmacology in humans. As in the 384-well format, identical 3D morphology of four pancreatic cancer-associated cells and comparable CRC of five control compounds were recapitulated in the 1536-well format. Future efforts will be focused on the screening of larger chemical libraries (~150,000) in a 1536-well automated platform and exploring 3D pancreatic cancer/CAF coculture models to better predict the response of drugs to the treatment of patients with pancreatic cancer. We will also follow up in mouse models of pancreatic tumors.

## Supplementary Material

Supplementary material

Supplementary material
