# Supplementary material for: Advanced Development of Primary Pancreatic Organoid Tumor Models for High-Throughput Phenotypic Drug Screening
Source: SLAS Discov. 2018 Apr 19;23(6):574–84. doi: 10.1177/2472555218766842 (PMC6013403; doi:10.1177/2472555218766842)
Supplement: Supplementary material [file Supp_Mat_for_Development_of_Primary_Pancreatic_Organoid_Tumor_Models_for_HT_Pheno_Drug_Screening_by_Hue_et_al.pdf]

Advanced Development of Primary Pancreatic Organoid Tumor Models for High Throughput Phenotypic Drug Screening

Shurong Hou[1γ], Hervé Triac[2γ], Banu Priya Sridharan[1], Louis Scampavia[1], Franck Madoux[1†], Jan Seldin[3], Glauco R. Souza[4], Donald Watson[5], David Tuveson[2] and Timothy P. Spicer[1*]

[1]The Scripps Research Institute Molecular Screening Center, Department of Molecular Medicine, Scripps Florida, 130 Scripps Way, Jupiter, Florida, USA

[2]Cancer Center, Cold Spring Harbor Laboratory, One Bungtown Road, Cold Spring Harbor, New York, USA

[3]Greiner Bio-One North America Inc., 4238 Capital Drive, Monroe, NC, USA

[4] Nano3D Biosciences, Inc. and University of Texas Health Science Center at Houston, Houston Texas, 7000 Fannin St., Houston TX, USA

[5]Dana-Farber Cancer Institute, 450 Brookline Ave., BA464, Boston MA, USA

[γ]Equal contribution

[†]Currently at Amgen Inc., Thousand Oaks, CA, USA

*Co-communicated by DT and TS

Short title:

Development of 3D Tumor Models for High-throughput Screening

Key words: Organoid, Pancreatic, Cancer, Phenotypic, HTS

*Address correspondence to:
Timothy Spicer
Scripps Florida
130 Scripps Way #1A1
Jupiter, FL 33458
U.S.A.
Email: spicert@scripps.edu
561-228-2150

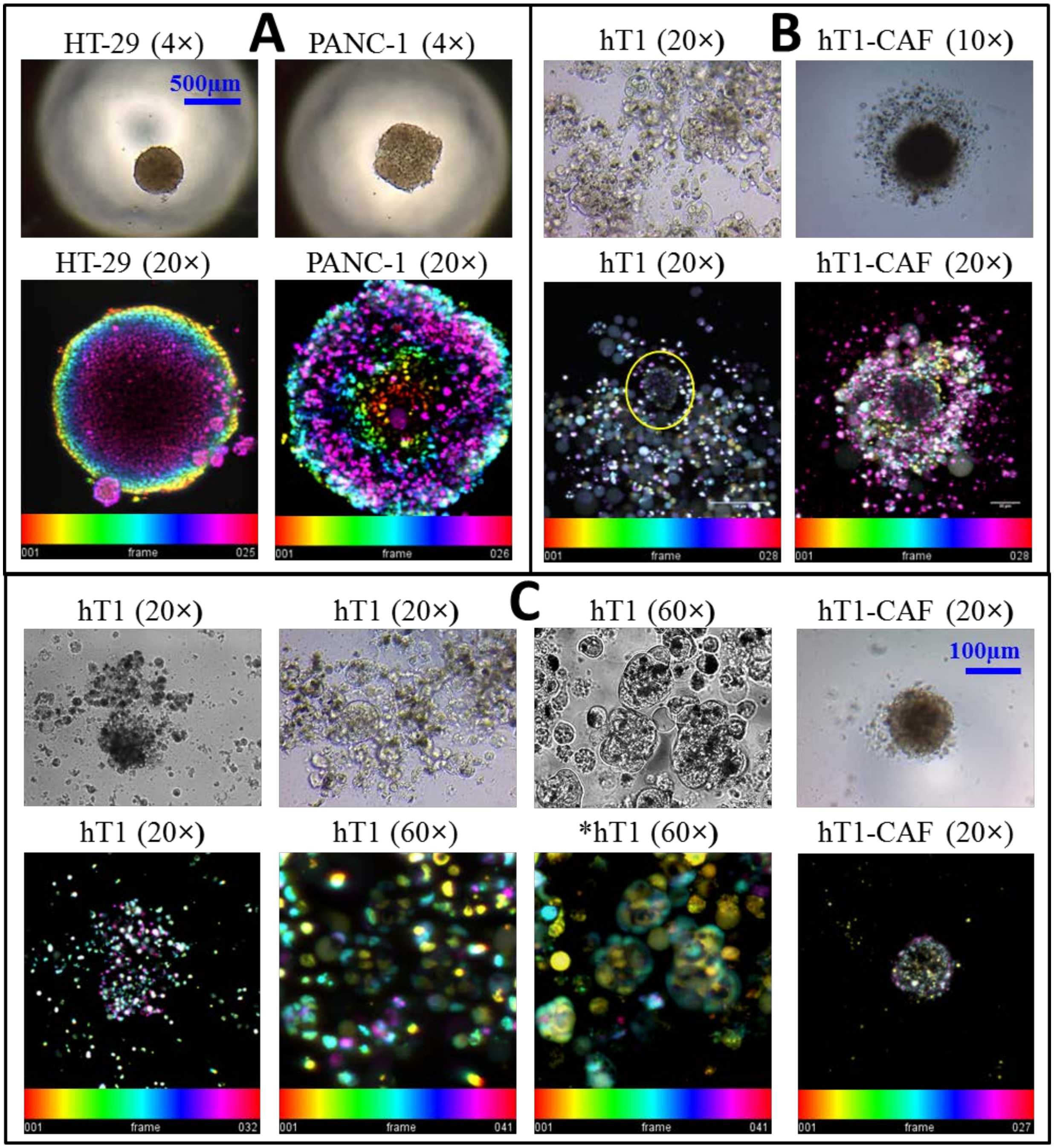

Supplemental Figure 1. 3D morphology of primary pancreatic cancer cells, associated fibroblasts and standard cell lines cultured using n3D bioprinting technology, imaged with standard microscopy (top) and INCell 6000 confocal microscopy (bottom). Z-stack images were obtained by nuclei staining with Hoechst or CellTracker Green staining then 2 or 5 micron image slices

with confocal microscopy and stacked using ImageJ software. HT-29 and PANC-1 spheroids were used as standards **(A)**. Wide field and Z-stack images of hT1 and hT1-CAF 3D culture in 384w and 1536w plates were presented in **(B)** and **(C),** respectively. Note: Z-stack image of hT1 organoids at 60× objective that marked with * was stained with CellTracker Green dye. Morphology of another pair of pancreatic cancer and cancer associated fibroblasts (hM1 and hM1-CAF, images not shown here) are similar to hT1 and hT1-CAF. Z-stack images confirmed 3D structure formation of primary pancreatic cancer cells and their associated fibroblasts in both 384w and 1536w plates.

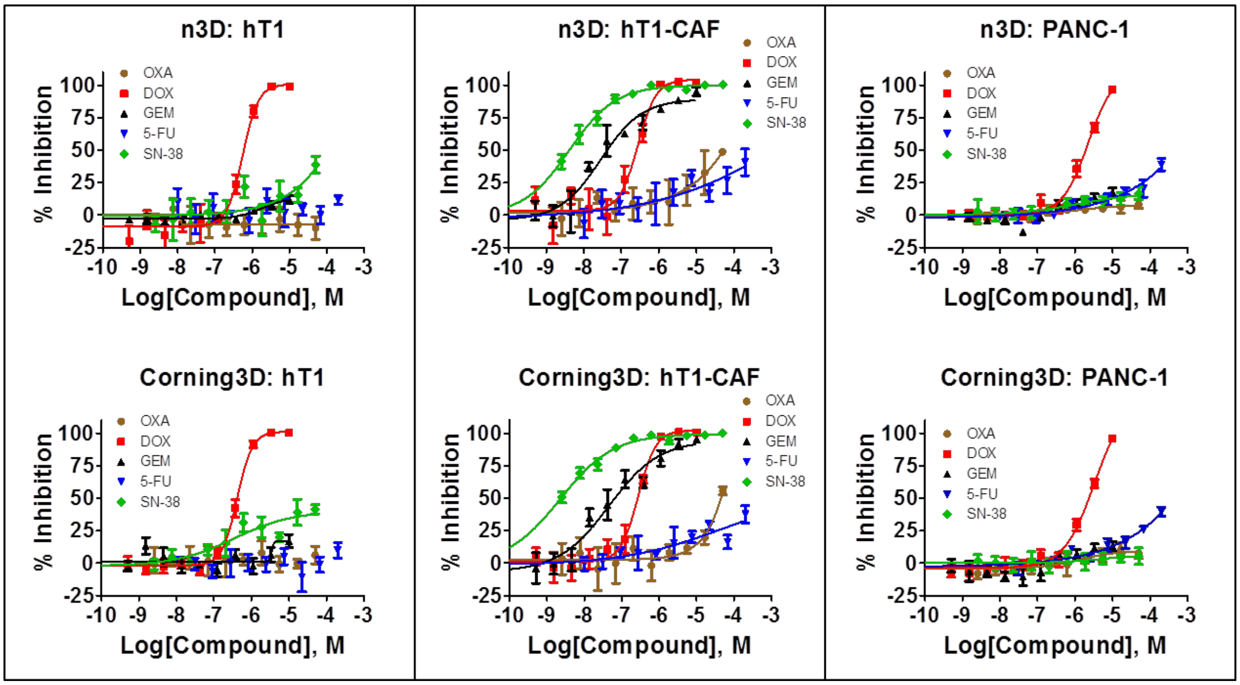

Supplemental Figure 2. Concentration-response curves for 5 control compounds (Oxaliplatin, Doxorubicin, Gemcitabine, 5-Fluorouracil and SN-38) vs. hT1, hT1-CAF and PANC-1 in 3D formats using n3D bioprinting technology and Corning spheroid plate. Each curve represents the

mean and standard deviation of 4 replicates in 384w. The control drugs produced identical responses on each 3D cell models using these two 3D culture technologies.

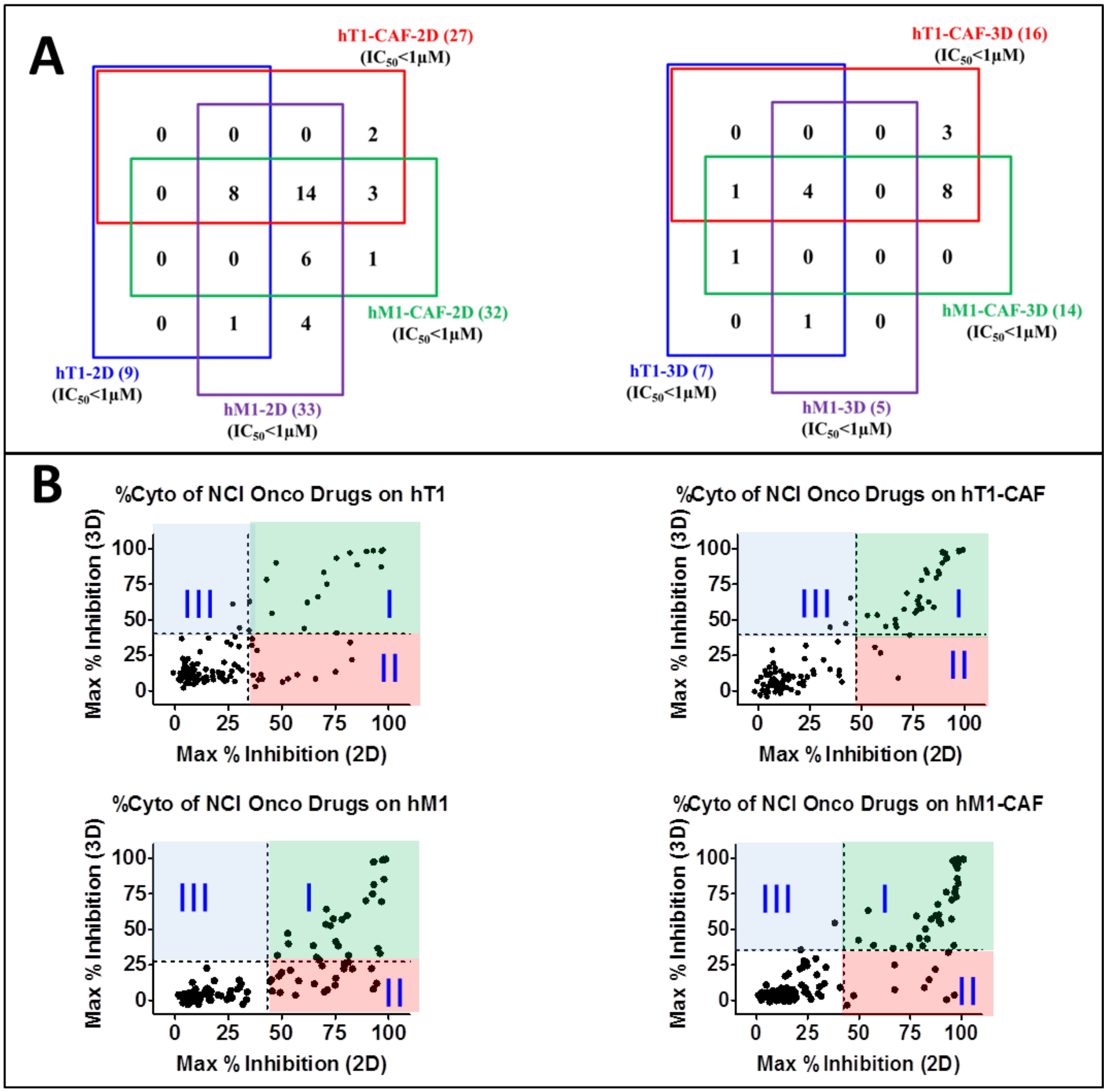

Supplemental Figure 3. Titration results of 114 NCI oncology drugs vs. 2D and 3D of CAFs or pancreatic cancer cells. (A) Four-way Venn diagram of active compounds identified from titration assays of 114 NCI oncology drugs in 2D (left) and 3D (right) models of CAFs and cancer cells

(hT1, hT1-CAF, hM1, hM1-CAF). An active compound was defined as the compound with an $IC_{50} < 1$ µM. The numbers in parentheses next to the target are the numbers of active compounds specific for that cell line. The numbers in the box represent the number of compounds found to active in those overlapping assays. (B) The correlation plot of the maximum % cytotoxicity values of the NCI oncology drugs in the 3D and 2D models of each of the 4 pancreatic cancer-associated cells. The dashed lines represent the hit-cutoffs derived from the HTS against the approved drug library (see supplemental figure 4 below).

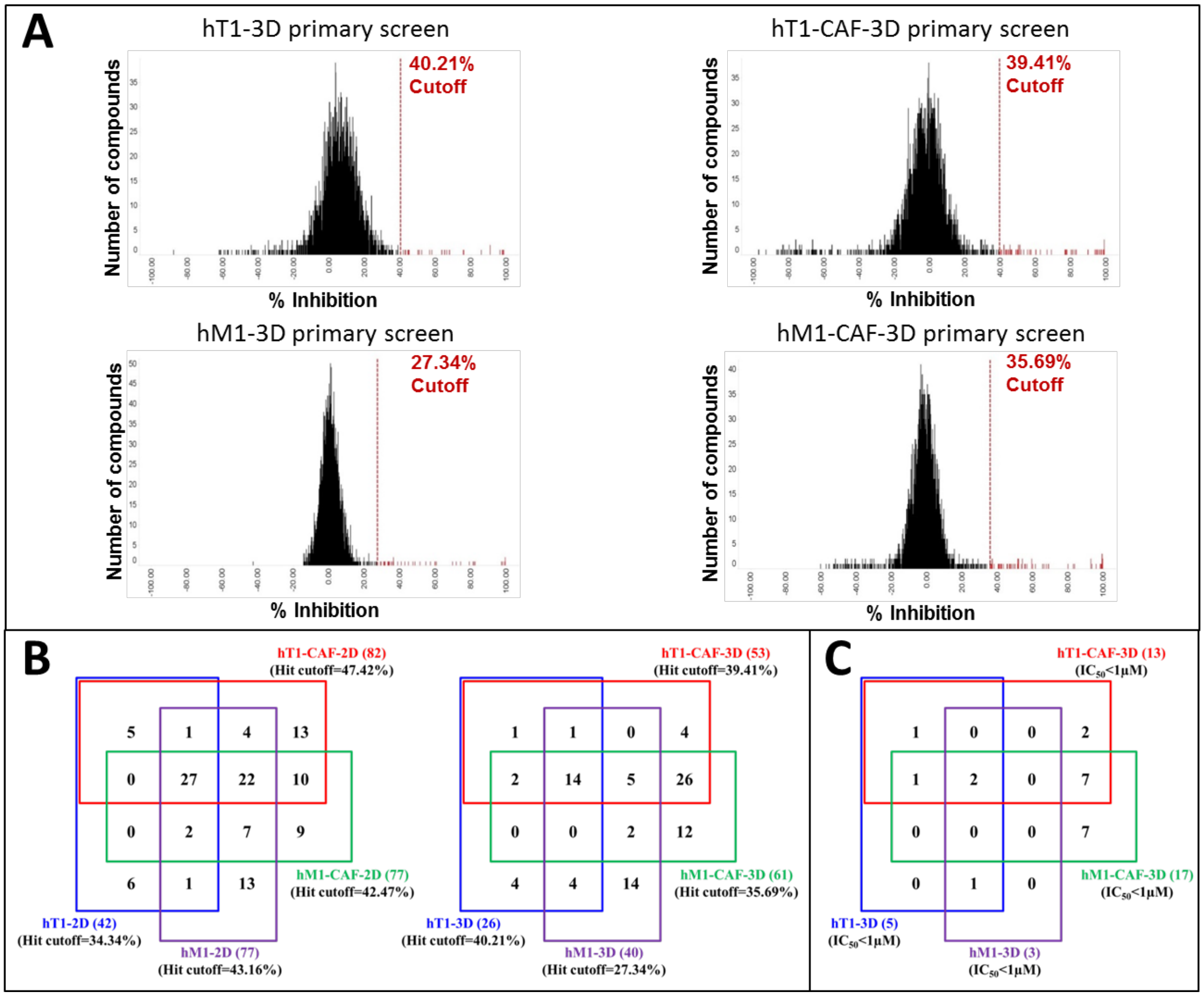

Supplemental Figure 4. Primary screen and titration results of 3,290 approved drugs vs. 2D and 3D of CAFs or pancreatic cancer cells. (A) Activity histogram of 3,290 approved drugs in each 3D primary screen, with corresponding hit cutoff shown in each plot. (B) Four-way Venn diagram of hits identified from primary screen in 2D (left) and 3D (right) models of CAFs and cancer cells (hT1, hT1-CAF, hM1, hM1-CAF). A hit was identified as any compound with % inhibition > the corresponding screen hit cutoff. The numbers in parentheses next to the target are the numbers of hits specific for that cell line. The numbers in the box represent the number of compounds found to active in those overlapping assays. (C) Four-way Venn diagram of active compounds (IC_{50} < 1 μM) identified from titration assays of 57 inhibitory compounds in 3D models of CAFs and cancer cells (hT1, hT1-CAF, hM1, hM1-CAF).

Supplemental Table 1. Titration results of 114 NCI oncology drugs vs. 2D and 3D of CAFs or pancreatic cancer cells demonstrating each drugs activity associated with its sample ID, drug name, logIC50, and the average maximum % response achieved for each drug. Note, typically the average maximum % response was found at the highest concentration tested but may not be the case for all drugs tested.

Sample ID	Drug Name	hT1-3D Final IC50	hT1-3D Averaged Max % Response	hT1-CAF-3D Final IC50	hT1-CAF-3D Averaged Max % Response	hM1-3D Final IC50	hM1-3D Averaged Max % Response	hM1-CAF-3D Final IC50	hM1-CAF-3D Averaged Max % Response	hT1-2D Final IC50	hT1-2D Averaged Max % Response	hT1-CAF-2D Final IC50	hT1-CAF-2D Averaged Max % Response	hM1-2D Final IC50	hM1-2D Averaged Max % Response	hM1-CAF-2D Final IC50	hM1-CAF-2D Averaged Max % Response
SR-01000941582-1	Carfilzomib	= 55.1E-9	99.24	= 83.4E-9	99.30	= 63.1E-9	99.49	= 74.3E-9	99.54	= 16.6E-9	97.35	= 10.3E-9	99.35	= 19.5E-9	98.69	= 10.2E-9	100.43
SR-01000597562-2	Homoharringtonine	= 333.7E-9	98.74	= 174.7E-9	98.17	= 155.9E-9	98.83	= 140.6E-9	99.31	= 182.2E-9	92.89	= 19.2E-9	97.35	= 16.7E-9	97.22	= 10.3E-9	101.05
SR-01000939863-2	Bortezomib	= 41.1E-9	98.58	= 59.3E-9	98.95	= 128.1E-9	99.26	= 38.5E-9	99.48	= 10.3E-9	96.63	= 10.E-9	97.17	= 16.2E-9	97.84	= 8.7E-9	98.21
SR-01000003049-13	Doxorubicin	= 1.5E-6	98.28	= 993.E-9	98.21	= 1.7E-6	97.68	= 1.4E-6	99.19	= 956.4E-9	89.77	= 535.E-9	89.28	= 212.E-9	93.20	= 144.1E-9	97.37
SR-01000000033-10	Daunorubicin	= 3.7E-6	97.28	= 2.2E-6	97.04	= 3.4E-6	97.48	= 2.3E-6	98.22	= 1.9E-6	81.97	= 638.E-9	90.91	= 178.E-9	92.92	= 42.E-9	95.81
SR-05000002335-3	Plicamycin	= 661.6E-9	93.51	= 313.6E-9	97.18	= 1.2E-6	81.44	= 388.9E-9	98.66	= 73.E-9	75.65	= 66.3E-9	89.53	= 18.7E-9	93.11	= 18.7E-9	96.42
SR-01000941589-1	Trametinib	= 4.7E-9	90.15	= 1.4E-6	65.42	= 16.5E-9	64.27	= 2.8E-6	54.55	> 5.E-6	47.37	> 5.E-6	44.82	= 2.7E-9	70.89	> 5.E-6	38.20
SR-01000941567-1	Ponatinib	= 481.6E-9	88.69	= 1.E-6	92.62	= 3.6E-6	59.73	= 681.E-9	96.94	= 1.2E-6	85.38	= 750.7E-9	90.81	= 1.2E-6	80.68	= 821.3E-9	98.45
SR-01000941579-1	Romidepsin	= 19.E-9	87.42	= 1.8E-9	98.97	= 46.E-9	85.36	= 3.5E-9	99.36	= 790.9E-12	96.62	= 538.4E-12	98.12	= 1.6E-9	97.89	= 2.4E-9	98.64
SR-01000763557-5	Epirubicin	= 2.9E-6	83.68	= 5.2E-6	85.33	= 3.5E-6	69.50	= 2.9E-6	78.91	= 3.5E-6	69.67	= 742.8E-9	81.27	= 194.7E-9	96.81	= 131.1E-9	97.05
SR-00000000005-2	Sunitinib	= 1.8E-6	78.44	> 6.6E-6	45.04	> 6.6E-6	31.78	> 6.6E-6	29.48	> 5.E-6	42.93	= 2.E-6	67.10	> 5.E-6	47.89	> 5.E-6	28.99
SR-01000879991-2	Valrubicin	= 4.5E-6	75.17	= 2.5E-6	93.37	= 4.8E-6	75.01	= 2.8E-6	93.25	= 3.2E-6	71.15	= 1.E-6	91.19	= 884.9E-9	92.56	= 748.E-9	98.02
SR-00000000462-2	Vandetanib	= 3.E-6	66.34	= 5.6E-6	63.41	> 6.6E-6	41.47	> 6.6E-6	38.28	= 2.9E-6	66.95	= 3.9E-6	77.33	= 524.5E-9	75.29	= 2.1E-6	74.74
SR-05000001460-5	Erlotinib	> 2.2E-6	63.00	> 6.6E-6	21.86	= 3.6E-6	53.60	> 6.6E-6	22.28	> 5.E-6	35.00	> 5.E-6	31.67	= 648.4E-9	70.73	> 5.E-6	21.28
SR-01000941576-1	Afatinib	= 4.4E-6	62.32	= 5.7E-6	60.98	> 2.2E-6	57.05	> 6.6E-6	33.81	> 1.7E-6	61.94	= 1.7E-6	77.72	= 226.3E-9	78.23	= 2.8E-6	93.66
SR-01000941575-1	Crizotinib	= 3.4E-6	61.23	= 4.9E-6	68.93	> 6.6E-6	28.43	> 6.6E-6	38.78	> 5.E-6	27.16	= 2.6E-6	72.13	= 2.1E-6	67.70	= 3.9E-6	57.08
SR-01000076001-15	Mitoxantrone	= 4.8E-6	54.79	= 1.7E-6	62.75	> 6.6E-6	22.22	= 1.4E-6	57.27	> 5.E-6	45.45	= 310.2E-9	82.74	= 2.1E-6	83.09	= 86.5E-9	95.10
SR-01000941569-1	Cabozantinib	> 6.6E-6	44.34	> 6.6E-6	30.86	> 6.6E-6	1.81	> 6.6E-6	42.58	> 5.E-6	30.22	= 4.5E-6	56.65	> 5.E-6	26.69	> 5.E-6	49.78
SR-01000941572-1	Bosutinib	> 6.6E-6	43.82	> 6.6E-6	22.63	= 3.6E-6	57.57	> 6.6E-6	4.74	= 2.E-6	60.48	> 5.E-6	22.63	= 1.2E-6	74.09	> 5.E-6	30.13
SR-01000941585-1	Cabazitaxel	> 6.6E-6	42.77	= 1.4E-9	56.81	> 6.6E-6	24.43	= 5.3E-9	57.14	> 5.E-6	34.69	< 253.5E-12	77.03	= 1.E-9	79.29	< 253.5E-12	85.25
SR-00000000554-4	Dasatinib	> 6.6E-6	40.95	> 2.2E-6	53.43	> 6.6E-6	31.68	> 6.6E-6	43.74	= 134.5E-9	75.70	= 537.8E-9	57.69	= 45.6E-9	81.28	= 240.1E-9	79.49
SR-01000941592-1	Everolimus	> 6.6E-6	38.26	> 6.6E-6	44.94	> 6.6E-6	11.80	> 6.6E-6	26.66	> 5.E-6	28.16	> 5.E-6	34.99	> 5.E-6	31.00	> 5.E-6	24.54
SR-00000000262-5	Gefitinib	> 6.6E-6	37.24	> 6.6E-6	28.87	> 6.6E-6	47.26	> 6.6E-6	7.40	> 5.E-6	15.78	> 5.E-6	6.97	> 1.7E-6	52.76	> 5.E-6	15.86
SR-01000941566-1	Axitinib	> 6.6E-6	36.85	= 5.1E-6	53.05	> 6.6E-6	7.24	= 1.8E-6	63.36	> 5.E-6	3.17	= 2.E-6	53.02	> 5.E-6	14.38	= 2.1E-6	54.52
SR-01000003023-8	Docetaxel	> 6.6E-6	36.80	= 5.6E-9	55.01	> 6.6E-6	26.09	= 4.8E-9	59.97	> 5.E-6	36.17	= 581.3E-12	76.12	= 1.E-9	79.44	= 508.6E-12	87.41
SR-01000941591-1	Sirolimus	> 6.6E-6	34.35	> 6.6E-6	34.63	> 6.6E-6	7.98	> 6.6E-6	23.81	> 5.E-6	24.41	> 5.E-6	38.63	> 5.E-6	29.30	> 5.E-6	23.12
SR-01000597410-2	Dactinomycin	> 6.6E-6	34.16	= 280.8E-9	92.97	> 6.6E-6	36.77	= 441.E-9	85.88	= 70.6E-9	82.03	= 39.8E-9	91.42	= 8.8E-9	95.05	= 15.9E-9	97.89
SR-05000001472-4	Lapatinib	> 6.6E-6	32.76	> 6.6E-6	15.14	> 6.6E-6	21.24	> 6.6E-6	8.53	> 5.E-6	26.11	> 5.E-6	34.86	= 3.2E-6	54.17	> 5.E-6	24.02
SR-01000941577-1	Ixabepilone	> 6.6E-6	32.17	= 580.2E-9	58.04	> 6.6E-6	26.94	= 593.2E-9	60.30	> 5.E-6	35.94	= 63.8E-9	79.38	= 12.2E-9	81.23	= 14.9E-9	88.98
SR-01000898799-2	Temsirolimus	> 6.6E-6	31.24	> 6.6E-6	47.55	> 6.6E-6	14.29	> 6.6E-6	28.09	> 5.E-6	29.94	> 5.E-6	42.55	> 5.E-6	30.01	> 5.E-6	23.14
SR-01000075350-9	Taxol	> 6.6E-6	28.53	= 63.8E-9	58.01	> 6.6E-6	22.14	= 329.6E-9	54.27	> 5.E-6	38.37	= 6.8E-9	78.64	= 12.2E-9	79.08	= 8.9E-9	89.88
SR-01000941573-1	Pomalidomide	> 6.6E-6	27.52	> 6.6E-6	16.62	> 6.6E-6	12.73	> 6.6E-6	8.87	> 5.E-6	11.78	> 5.E-6	5.31	> 5.E-6	8.15	> 5.E-6	14.70
SR-01000597411-6	Methotrexate	> 6.6E-6	22.84	> 6.6E-6	10.80	> 6.6E-6	10.77	> 6.6E-6	7.52	> 5.E-6	3.71	> 5.E-6	14.45	= 74.5E-9	74.94	= 184.5E-9	67.45
SR-01000759347-8	Temozolomide	> 6.6E-6	22.68	> 6.6E-6	5.13	> 6.6E-6	2.38	> 6.6E-6	5.20	> 5.E-6	6.03	> 5.E-6	5.86	> 5.E-6	8.45	> 5.E-6	16.62
SR-01000075934-8	Idarubicin	> 6.6E-6	22.03	= 6.5E-6	58.80	> 6.6E-6	12.17	> 6.6E-6	38.73	= 3.7E-6	82.84	= 1.9E-6	85.17	= 81.8E-9	94.48	= 7.5E-9	96.32
SR-01000075919-8	Hydroxyurea	> 6.6E-6	20.78	> 6.6E-6	9.29	> 6.6E-6	4.27	> 6.6E-6	4.48	> 5.E-6	4.89	> 5.E-6	13.00	> 5.E-6	14.94	> 5.E-6	25.25
SR-01000000155-6	Vinblastine	> 6.6E-6	20.15	> 243.8E-9	50.24	> 6.6E-6	38.62	> 6.6E-6	43.37	> 5.E-6	23.06	= 77.8E-9	66.58	> 6.8E-9	64.85	= 2.5E-9	83.37
SR-01000075736-7	Carmustine	> 6.6E-6	19.86	> 6.6E-6	2.86	> 6.6E-6	5.40	> 6.6E-6	5.09	> 5.E-6	4.72	> 5.E-6	9.50	> 5.E-6	6.76	> 5.E-6	7.20
SR-05000002022-4	Ifosfamide	> 6.6E-6	19.11	> 6.6E-6	-1.76	> 6.6E-6	-2.61	> 6.6E-6	-0.80	> 5.E-6	10.52	> 5.E-6	3.94	> 5.E-6	31.82	> 5.E-6	16.04
SR-01000941581-1	Estramustine phosphate	> 6.6E-6	18.67	> 6.6E-6	5.04	> 6.6E-6	3.92	> 6.6E-6	2.38	> 5.E-6	8.22	> 5.E-6	11.98	> 5.E-6	10.71	> 5.E-6	16.93
SR-01000941588-1	Vinorelbine	> 6.6E-6	18.59	= 318.4E-9	57.56	> 6.6E-6	24.36	= 508.1E-9	55.73	> 5.E-6	27.91	= 174.7E-9	70.85	= 246.6E-9	68.88	= 172.6E-9	89.03
SR-01000883999-3	Lenalidomide	> 6.6E-6	17.73	> 6.6E-6	6.89	> 6.6E-6	0.31	> 6.6E-6	4.75	> 5.E-6	4.79	> 5.E-6	-0.45	> 5.E-6	16.11	> 5.E-6	8.94
SR-01000000117-11	Altretamine	> 6.6E-6	17.09	> 6.6E-6	10.80	> 6.6E-6	3.89	> 6.6E-6	5.64	> 5.E-6	5.31	> 5.E-6	14.52	> 5.E-6	20.22	> 5.E-6	18.95
SR-01000000186-9	Vincristine	> 6.6E-6	16.82	> 6.6E-6	39.25	> 6.6E-6	39.81	> 6.6E-6	38.32	> 5.E-6	29.55	= 176.7E-9	73.49	> 6.8E-9	53.13	= 69.7E-9	80.95
SR-01000883995-4	Dexrazoxane	> 6.6E-6	15.89	> 6.6E-6	10.88	> 6.6E-6	5.77	> 6.6E-6	4.53	> 5.E-6	4.16	> 5.E-6	12.56	> 5.E-6	8.73	> 5.E-6	10.55
SR-05000001855-4	Thiotepa	> 6.6E-6	15.74	> 6.6E-6	7.92	> 6.6E-6	2.91	> 6.6E-6	3.07	> 5.E-6	5.83	> 5.E-6	12.53	> 5.E-6	2.25	> 5.E-6	31.49
SR-01000941584-1	Abiraterone	> 6.6E-6	15.44	> 6.6E-6	0.60	> 6.6E-6	3.99	> 6.6E-6	2.07	> 5.E-6	4.90	> 5.E-6	2.16	> 5.E-6	1.77	> 5.E-6	10.25
SR-01000000062-11	Chlorambucil	> 6.6E-6	15.27	> 6.6E-6	6.74	> 6.6E-6	4.48	> 6.6E-6	4.61	> 5.E-6	14.46	> 5.E-6	22.04	> 5.E-6	15.50	> 5.E-6	18.35

SR-01000629727-7	Methoxsalen	>	6.6E-6	15.12	>	6.6E-6	14.21	>	6.6E-6	13.83	>	6.6E-6	6.87	>	5.E-6	7.00	>	5.E-6	3.39	>	5.E-6	18.44	>	5.E-6	8.06
SR-05000001845-7	Mitomycin	>	6.6E-6	15.00	>	6.6E-6	16.32	>	6.6E-6	8.03	>	6.6E-6	3.74	>	5.E-6	5.08	>	5.E-6	8.28	=	817.9E-9	92.86	=	184.9E-9	96.56
SR-01000930807-2	Uracil mustard	>	6.6E-6	14.93	>	6.6E-6	11.86	>	6.6E-6	6.92	>	6.6E-6	18.09	>	5.E-6	9.09	>	5.E-6	26.95	>	5.E-6	26.07	>	5.E-6	29.52
SR-01000721860-8	Cytarabine	>	6.6E-6	14.48	>	6.6E-6	26.77	>	6.6E-6	17.14	>	6.6E-6	9.13	>	5.E-6	31.38	=	960.3E-9	59.36	>	5.E-6	48.71	=	1.7E-6	81.90
SR-01000000001-6	Floxuridine	>	6.6E-6	13.98	>	6.6E-6	14.42	>	6.6E-6	7.43	>	6.6E-6	14.70	>	5.E-6	26.64	>	5.E-6	39.41	>	61.6E-9	71.34	=	192.9E-9	84.26
SR-01000075881-7	Fluorouracil	>	6.6E-6	13.92	>	6.6E-6	9.99	>	6.6E-6	6.69	>	6.6E-6	11.98	>	5.E-6	17.55	>	5.E-6	17.00	>	5.E-6	45.58	>	5.E-6	33.16
SR-05000000373-5	Vorinostat	>	6.6E-6	13.44	=	1.4E-6	90.06	=	2.5E-6	52.70	=	4.6E-6	67.53	=	2.1E-6	75.23	=	1.1E-6	89.23	=	2.3E-6	72.74	=	1.7E-6	88.47
SR-01000883935-2	Pentostatin	>	6.6E-6	13.21	>	6.6E-6	13.91	>	6.6E-6	3.90	>	6.6E-6	7.67	>	5.E-6	8.56	>	5.E-6	11.11	>	5.E-6	12.61	>	5.E-6	17.72
SR-01000076102-14	Raloxifene	>	6.6E-6	13.20	>	6.6E-6	2.11	>	6.6E-6	3.97	>	6.6E-6	2.14	>	5.E-6	20.33	>	5.E-6	9.69	>	5.E-6	8.97	>	5.E-6	17.13
SR-01000941580-1	Enzalutamide	>	6.6E-6	13.01	>	6.6E-6	9.92	>	6.6E-6	6.66	>	6.6E-6	0.95	>	5.E-6	6.28	>	5.E-6	23.81	>	5.E-6	9.09	>	5.E-6	11.31
SR-01000931150-2	Nilotinib	>	6.6E-6	12.99	>	6.6E-6	0.67	>	6.6E-6	0.45	>	6.6E-6	2.22	>	5.E-6	8.40	>	5.E-6	4.15	>	5.E-6	5.88	>	5.E-6	3.92
SR-01000759390-7	Anastrozole	>	6.6E-6	12.72	>	6.6E-6	-4.00	>	6.6E-6	1.33	>	6.6E-6	1.97	>	5.E-6	8.23	>	5.E-6	4.30	>	5.E-6	11.57	>	5.E-6	8.59
SR-01000000239-22	Tretinoin	>	6.6E-6	12.71	>	6.6E-6	7.89	>	6.6E-6	5.82	>	6.6E-6	1.90	>	5.E-6	5.65	>	5.E-6	14.54	>	5.E-6	14.65	>	5.E-6	19.47
SR-05000001664-2	Mechlorethamine	>	6.6E-6	12.68	>	6.6E-6	11.23	>	6.6E-6	3.41	>	6.6E-6	0.87	>	5.E-6	2.45	>	5.E-6	5.49	>	5.E-6	25.91	>	5.E-6	26.65
SR-01000942221-2	Arsenic trioxide	>	6.6E-6	12.68	>	6.6E-6	-0.78	>	6.6E-6	-1.68	>	6.6E-6	5.54	>	5.E-6	-0.84	>	5.E-6	8.27	>	5.E-6	3.77	>	5.E-6	2.58
SR-05000001858-2	Pipobroman	>	6.6E-6	12.47	>	6.6E-6	3.40	>	6.6E-6	-1.98	>	6.6E-6	3.11	>	5.E-6	3.71	>	5.E-6	4.83	>	5.E-6	6.63	>	5.E-6	6.76
SR-01000075523-19	Tamoxifen	>	6.6E-6	12.39	>	6.6E-6	31.93	>	6.6E-6	22.80	>	6.6E-6	17.11	>	5.E-6	22.30	>	5.E-6	23.14	>	5.E-6	14.93	>	5.E-6	14.02
SR-01000838879-3	Decitabine	>	6.6E-6	11.85	>	6.6E-6	10.45	>	6.6E-6	6.43	>	6.6E-6	8.56	>	5.E-6	4.05	>	5.E-6	15.43	>	5.E-6	16.89	>	5.E-6	12.12
SR-01000939639-2	Pazopanib	>	6.6E-6	11.65	>	6.6E-6	45.49	>	6.6E-6	0.73	=	3.9E-6	59.41	>	5.E-6	11.12	=	3.E-6	61.97	>	5.E-6	24.37	=	2.3E-6	77.99
SR-01000941578-1	Pralatrexate	>	6.6E-6	11.55	>	6.6E-6	14.49	>	6.6E-6	15.64	>	6.6E-6	25.16	>	5.E-6	40.23	>	5.E-6	26.69	<	253.5E-12	75.32	=	2.7E-9	67.37
SR-01000775528-3	Teniposide	>	6.6E-6	11.46	=	602.5E-9	82.43	>	6.6E-6	22.40	=	787.1E-9	76.23	=	1.7E-6	57.33	=	235.9E-9	87.83	=	893.1E-9	92.10	=	305.5E-9	92.56
SR-01000941586-1	Bleomycin	>	6.6E-6	11.30	=	1.3E-6	79.66	>	6.6E-6	14.58	=	1.3E-6	76.22	>	5.E-6	36.66	=	569.8E-9	86.37	>	5.E-6	45.25	=	317.6E-9	96.77
SR-01000075680-7	Amifostine	>	6.6E-6	11.17	>	6.6E-6	3.81	>	6.6E-6	-2.85	>	6.6E-6	1.43	>	5.E-6	3.54	>	5.E-6	6.73	>	5.E-6	11.00	>	5.E-6	8.17
SR-05000001497-2	Lomustine	>	6.6E-6	11.14	>	6.6E-6	2.63	>	6.6E-6	-0.30	>	6.6E-6	1.83	>	5.E-6	6.45	>	5.E-6	10.99	>	5.E-6	5.54	>	5.E-6	5.82
SR-01000759393-6	Exemestane	>	6.6E-6	11.11	>	6.6E-6	11.76	>	6.6E-6	3.39	>	6.6E-6	2.98	>	5.E-6	8.37	>	5.E-6	5.02	>	5.E-6	9.68	>	5.E-6	14.47
SR-01000076184-13	Thalidomide	>	6.6E-6	10.98	>	6.6E-6	9.62	>	6.6E-6	3.34	>	6.6E-6	3.67	>	5.E-6	8.89	>	5.E-6	1.38	>	5.E-6	3.01	>	5.E-6	0.98
SR-01000941587-1	Triethylenemelamine	>	6.6E-6	10.91	>	6.6E-6	8.20	>	6.6E-6	5.54	>	6.6E-6	0.53	>	5.E-6	8.05	>	5.E-6	6.78	>	5.E-6	49.22	=	1.2E-6	92.69
SR-01000939745-2	Streptozocin	>	6.6E-6	10.64	>	6.6E-6	12.25	>	6.6E-6	3.75	>	6.6E-6	6.09	>	5.E-6	7.46	>	5.E-6	5.95	>	5.E-6	9.00	>	5.E-6	11.57
SR-01000941574-1	Vismodegib	>	6.6E-6	10.53	>	6.6E-6	8.69	>	6.6E-6	4.75	>	6.6E-6	10.97	>	5.E-6	6.26	>	5.E-6	7.60	>	5.E-6	9.43	>	5.E-6	4.77
SR-02000000903-2	Bendamustine	>	6.6E-6	10.51	>	6.6E-6	2.42	>	6.6E-6	-0.46	>	6.6E-6	0.36	>	5.E-6	2.85	>	5.E-6	13.73	>	5.E-6	8.56	>	5.E-6	2.38
SR-01000075662-13	Azacitidine	>	6.6E-6	10.37	>	6.6E-6	11.07	>	6.6E-6	13.41	>	6.6E-6	9.61	>	5.E-6	17.62	>	5.E-6	39.28	>	5.E-6	44.59	>	5.E-6	30.85
SR-01000003063-10	Cladribine	>	6.6E-6	10.29	=	1.2E-6	77.80	>	6.6E-6	38.32	=	2.2E-6	70.85	>	5.E-6	25.99	=	720.2E-9	79.25	=	550.E-9	76.50	=	669.6E-9	95.31
SR-01000597471-4	Fulvestrant	>	6.6E-6	9.88	>	6.6E-6	19.67	>	6.6E-6	8.41	>	6.6E-6	5.86	>	5.E-6	14.80	>	5.E-6	7.32	>	5.E-6	15.19	>	5.E-6	23.51
SR-01000763375-6	Procarbazine	>	6.6E-6	9.85	>	6.6E-6	6.39	>	6.6E-6	3.80	>	6.6E-6	8.34	>	5.E-6	20.97	>	5.E-6	11.94	>	5.E-6	16.36	>	5.E-6	14.88
SR-01000759382-8	Letrozole	>	6.6E-6	9.57	>	6.6E-6	-0.38	>	6.6E-6	-2.90	>	6.6E-6	1.90	>	5.E-6	2.81	>	5.E-6	-1.69	>	5.E-6	6.12	>	5.E-6	9.71
SR-01000075751-9	Mitotane	>	6.6E-6	9.41	>	6.6E-6	13.19	>	6.6E-6	2.52	>	6.6E-6	3.89	>	5.E-6	6.02	>	5.E-6	8.26	>	5.E-6	3.91	>	5.E-6	9.50
SR-01000075595-5	Allopurinol	>	6.6E-6	9.38	>	6.6E-6	-0.35	>	6.6E-6	0.28	>	6.6E-6	1.54	>	5.E-6	3.41	>	5.E-6	10.97	>	5.E-6	4.76	>	5.E-6	11.42
SR-01000941590-1	Dabrafenib	>	6.6E-6	8.67	>	6.6E-6	5.21	>	6.6E-6	0.56	>	6.6E-6	24.59	>	5.E-6	22.18	>	5.E-6	19.89	>	5.E-6	33.58	>	5.E-6	24.75
SR-01000931255-2	Capecitabine	>	6.6E-6	8.67	>	6.6E-6	4.31	>	6.6E-6	4.79	>	6.6E-6	5.76	>	5.E-6	8.32	>	5.E-6	10.60	>	5.E-6	25.68	>	5.E-6	13.30
SR-05000002138-6	Aminolevulinic acid	>	6.6E-6	8.60	>	6.6E-6	11.53	>	6.6E-6	8.02	>	6.6E-6	7.09	>	5.E-6	6.52	>	5.E-6	14.62	>	5.E-6	9.60	>	5.E-6	10.99
SR-05000001598-5	Dacarbazine	>	6.6E-6	8.55	>	6.6E-6	-0.57	>	6.6E-6	0.97	>	6.6E-6	1.72	>	5.E-6	12.95	>	5.E-6	4.96	>	5.E-6	10.40	>	5.E-6	5.79
SR-01000799142-7	Mercaptopurine	>	6.6E-6	8.54	>	6.6E-6	10.27	>	6.6E-6	6.02	>	6.6E-6	3.33	=	914.6E-9	52.99	>	5.E-6	20.78	=	862.2E-9	69.93	>	5.E-6	47.42
SR-01000763672-6	Topotecan	>	6.6E-6	8.53	=	1.4E-6	93.42	>	6.6E-6	33.03	=	1.8E-6	95.42	=	1.4E-6	65.88	=	374.9E-9	91.96	=	451.E-9	96.02	=	111.2E-9	97.23
SR-01000000258-8	Megestrol acetate	>	6.6E-6	8.48	>	6.6E-6	-3.01	>	6.6E-6	2.87	>	6.6E-6	0.80	>	5.E-6	6.55	>	5.E-6	1.01	>	5.E-6	3.44	>	5.E-6	13.13
SR-01000930565-2	Clofarabine	>	6.6E-6	8.46	=	544.1E-9	83.08	>	6.6E-6	30.43	=	2.E-6	73.06	>	5.E-6	40.95	=	277.6E-9	81.97	=	216.9E-9	66.21	=	339.E-9	97.49
SR-01000076253-5	Carboplatin	>	6.6E-6	8.04	>	6.6E-6	11.28	>	6.6E-6	3.50	>	6.6E-6	0.96	>	5.E-6	1.83	>	5.E-6	4.03	>	5.E-6	9.16	>	5.E-6	9.83
SR-01000941568-1	Vemurafenib	>	6.6E-6	8.00	>	6.6E-6	0.91	>	6.6E-6	0.42	>	6.6E-6	18.78	>	5.E-6	17.98	>	5.E-6	16.10	>	5.E-6	11.58	>	5.E-6	15.61
SR-01000075737-10	Cyclophosphamide	>	6.6E-6	7.88	>	6.6E-6	1.53	>	6.6E-6	3.32	>	6.6E-6	10.80	>	5.E-6	10.66	>	5.E-6	11.59	>	5.E-6	21.57	>	5.E-6	22.06
SR-05000001491-3	Gemcitabine	>	6.6E-6	7.79	=	240.4E-9	84.12	=	2.8E-6	70.22	=	172.9E-9	82.57	>	5.E-6	39.13	=	19.2E-9	87.30	=	34.9E-9	89.34	=	10.5E-9	98.50

SR-01000941571-1	Regorafenib	>	6.6E-6	7.73	>	6.6E-6	3.80	>	6.6E-6	19.64	>	6.6E-6	35.65	>	5.E-6	22.79	>	5.E-6	22.08	>	5.E-6	49.94	>	5.E-6	21.68
SR-01000941593-1	Plerixafor	>	6.6E-6	7.72	>	6.6E-6	5.86	>	6.6E-6	4.60	>	6.6E-6	-0.09	>	5.E-6	6.57	>	5.E-6	6.02	>	5.E-6	4.29	>	5.E-6	9.64
SR-01000837528-7	Celecoxib	>	6.6E-6	7.68	>	6.6E-6	9.39	>	6.6E-6	-0.32	>	6.6E-6	0.52	>	5.E-6	14.99	>	5.E-6	13.63	>	5.E-6	5.23	>	5.E-6	11.17
SR-01000763864-5	Irinotecan	>	6.6E-6	7.51	>	6.6E-6	47.07	>	6.6E-6	0.12	>	6.6E-6	36.63	>	5.E-6	24.07	=	2.6E-6	66.61	>	5.E-6	16.84	=	4.E-6	66.67
SR-01000075983-7	Melphalan	>	6.6E-6	7.41	>	6.6E-6	-1.13	>	6.6E-6	1.05	>	6.6E-6	9.12	>	5.E-6	6.30	>	5.E-6	19.24	>	5.E-6	9.53	>	5.E-6	40.91
SR-05000001436-2	Zoledronic acid	>	6.6E-6	7.40	>	6.6E-6	5.69	>	6.6E-6	1.08	>	6.6E-6	3.06	>	5.E-6	2.64	>	5.E-6	7.15	>	5.E-6	8.34	>	5.E-6	12.03
SR-01000765405-6	Busulfan	>	6.6E-6	7.38	>	6.6E-6	2.16	>	6.6E-6	2.72	>	6.6E-6	3.84	>	5.E-6	2.64	>	5.E-6	10.94	>	5.E-6	8.65	>	5.E-6	15.96
SR-00000000529-4	Sorafenib	>	6.6E-6	6.98	>	6.6E-6	8.46	>	6.6E-6	13.80	>	6.6E-6	23.39	>	5.E-6	23.36	>	5.E-6	14.97	=	2.4E-6	57.93	>	5.E-6	33.90
SR-05000001945-3	Fludarabine	>	6.6E-6	6.82	>	6.6E-6	6.34	>	6.6E-6	9.00	>	6.6E-6	5.70	>	5.E-6	28.06	>	5.E-6	40.73	>	5.E-6	30.26	>	5.E-6	5.22
SR-05000002077-4	Tioguanine	>	6.6E-6	6.39	>	6.6E-6	9.07	>	6.6E-6	11.62	>	6.6E-6	21.96	=	2.1E-6	50.29	=	2.2E-6	67.99	=	1.4E-6	65.22	=	758.1E-9	87.26
SR-01000931847-2	Nelarabine	>	6.6E-6	6.08	>	6.6E-6	4.64	>	6.6E-6	2.21	>	6.6E-6	-0.51	>	5.E-6	9.98	>	5.E-6	2.56	>	5.E-6	6.75	>	5.E-6	13.43
SR-01000611320-4	Imiquimod	>	6.6E-6	6.05	>	6.6E-6	6.25	>	6.6E-6	0.17	>	6.6E-6	0.48	>	5.E-6	13.68	>	5.E-6	9.25	>	5.E-6	9.08	>	5.E-6	18.43
SR-01000076254-5	Cisplatin	>	6.6E-6	5.46	>	6.6E-6	-1.63	>	6.6E-6	3.09	>	6.6E-6	2.74	>	5.E-6	6.79	>	5.E-6	10.76	>	5.E-6	4.45	>	5.E-6	13.11
SR-01000941570-1	Pemetrexed	>	6.6E-6	5.02	>	6.6E-6	6.07	>	6.6E-6	3.68	>	6.6E-6	-3.30	>	5.E-6	5.03	>	5.E-6	8.54	=	1.1E-6	56.44	>	5.E-6	44.25
SR-01000763561-8	Imatinib	>	6.6E-6	4.81	>	6.6E-6	8.72	>	6.6E-6	0.43	>	6.6E-6	-0.94	>	5.E-6	8.83	>	5.E-6	14.24	>	5.E-6	5.46	>	5.E-6	6.52
SR-01000763196-7	Etoposide	>	6.6E-6	3.10	=	3.3E-6	66.31	>	6.6E-6	5.91	=	6.5E-6	50.34	>	5.E-6	37.62	=	795.E-9	78.30	>	5.E-6	33.93	=	2.E-6	82.49
SR-01000780565-3	Oxaliplatin	>	6.6E-6	2.12	>	6.6E-6	1.73	>	6.6E-6	1.85	>	6.6E-6	2.69	>	5.E-6	3.97	>	5.E-6	8.97	>	5.E-6	10.49	>	5.E-6	11.38
