# Supplementary material for: Advanced Development of Primary Pancreatic Organoid Tumor Models for High-Throughput Phenotypic Drug Screening
Source: SLAS Discov. 2018 Apr 19;23(6):574–84. doi: 10.1177/2472555218766842 (PMC6013403; doi:10.1177/2472555218766842)
Supplement: Supplementary material [file DISC766842_OPEN_ACCESS_Signed_Form.pdf]

SAGE Choice
Contributor's Publishing Agreement

Please use Adobe Reader or Adobe Acrobat to complete your agreement

To be completed by the owner of copyright in the Contribution

**Title of Article**	: Advanced Development of Primary Pancreatic Organoid Tumor Models for High-Throughput Phenotypic Drug Screening
**Journal**	: SLAS Discovery
**All Author(s)**	: Shurong Hou, Hervé Tiriac, Banu Priya Sridharan, Louis Scampavia, Franck Madoux, Jan Seldin, Glauco R. Souza, Donald Watson, David Tuveson, and Timothy P. Spicer
**Corresponding Author**	: Timothy P. Spicer
**Corr. Author Address**	: Scripps Florida, 130 Scripps Way, Jupiter, FL 33458

Please read the notes attached, then complete, sign and return this Agreement to the **Journal's Production Editor**.

The author who signs this Agreement certifies that he/she is authorized to sign on behalf of him/herself and in the case of a multi-authored Contribution, on behalf of all other authors of the Contribution. *The authors understand that they each have the option of signing and returning a separate copy of this Agreement. This Agreement may be signed and executed by e-mail (a scanned hard copy of the Agreement with your signature on it or a digital original copy with your electronic signature are equally acceptable), by traditional hard copy or by fax.*

This Agreement will grant to the owner(s) (the 'Proprietor') of the Journal identified above (the Journal title subject to verification by SAGE Publishing ('SAGE')) a commercial license to produce, publish, sell and sub-license your article ('Article') and any accompanying abstract or Supplemental Material (all materials collectively referenced as the 'Contribution'), in all languages and all formats through any medium of communication for the full legal term of copyright (and any renewals) throughout the world. The Proprietor will publish the Contribution under the Creative Commons license selected by you below. Where the selected license is CC BY-NC or other Creative Commons non-commercial license, the Proprietor will receive exclusive commercial rights to the Article and non-exclusive commercial rights to the abstract and Supplemental Material.

Payment of the applicable Article Processing Charge (APC)* in the amount of **three thousand U.S. dollars ($3,000)** will be due (payable by the author or their funder or institution) prior to publication of the Contribution. Please contact openaccess@sagepub.com if you have any questions about the APC or any discounts that may be applicable to you.

**** Discounts/special journal pricing may apply:*** ______________________________ ***Promo Code:*** __________________

(If applicable, this section will be filled in by the Journal's Production Editor and is subject to final approval by SAGE)

Your institution may have an Open Access Prepaid Account with SAGE. If so, the author should enter their institutional Open Access Prepaid Account code below. In doing so the author warrants to SAGE that any necessary authorization for use of the code has been sought and confirmed.

Open Access Prepaid Account Code _________________________ (List of participating institutions)

Select ONE of the following:

See SAGE Publishing website for info on re-use of OA content: https://uk.sagepub.com/en-gb/eur/re-use-of-open-access-content

○ **Creative Commons Attribution Non-Commercial 4.0 license (CC BY NC 4.0)**
This license allows others to re-use the Contribution without permission as long as the Contribution is properly referenced and the use is non-commercial.

◉ **Creative Commons Attribution 4.0 License (CC BY 4.0) (if required by funding institution)**
This license allows others to re-use the Contribution without permission as long as the Contribution is properly referenced.

Some funding institutions require you to publish your research under this license. Please check the SAGE Open Access website (https://uk.sagepub.com/en-gb/eur/funding-bodies-policies-and-compliance) and indicate your funding information below. (All other funded authors should select the CC BY NC 4.0 license above.)

Funding Institution: NIH IMAT Award R33CA206949

By signing this Contributor Agreement you agree both to the above provisions and to the Terms of the Agreement attached below.

Contributor

Signature: Timothy P. Spicer Digitally signed by Timothy P. Spicer Date: 2018.03.21 18:52:17 -04'00' Date: 3/21/2018

If you are required to publish your research under a different license type, please contact openaccess@sagepub.com

You represent that the Contribution is owned by you unless one of the following is checked:

- ** If any author is an employee of the United States Government or of the Government of the country indicated below (non-U.S.) and prepared the Contribution as part of their official duties, please check here: ☐

  US Government Department or Agency Name: ____

  Non-US Government Department or Agency Name: ____

- If any author prepared the Contribution at the direction of their employer, please have a representative of your employer sign below, and please check here: ☐

  Employer Name: ____

  Authorized Signature: _______________________________ Date signed: _________________

****Government work.** If the Contribution was not prepared as part of the Contributor's official duties, it is not a Government work. If the Contribution was jointly authored, all the co-authors must have been Government employees at the time they prepared the Contribution in order for it to be a Government work; if any co-author was not a Government employee, then the Contribution is not a Government work. If the Contribution was prepared under a Government contract or grant, it is not a Government work - in such case, copyright is usually owned by the contractor or grantee.

TERMS OF THE AGREEMENT

Copyright.
While copyright remains yours as the author, you hereby authorise the Proprietor to act on your behalf to defend your copyright should it be infringed and to retain half of any damages awarded, after deducting costs.

Warranties.
You certify that:

- The Contribution is your original work and you have the right to enter into this Agreement and to convey the rights granted herein to the Proprietor.
- The Contribution is submitted for first publication in the Journal and is not being considered for publication elsewhere and has not already been published elsewhere, either in printed or electronic form (unless you has disclosed otherwise in writing to the Editor and approved by Editor).
- You have obtained and enclose all necessary permissions for the reproduction of any copyright works (e.g. quotes, photographs or other visual material, etc.) contained in the Contribution and not owned by you and that you have acknowledged all the source(s).
- The Contribution contains no violation of any existing copyright, other third party rights or any defamatory or untrue statements and does not infringe any rights of others.
- Any studies on which the Contribution is directly based were satisfactorily conducted in compliance with the governing Institutional Review Board (IRB) standards or were exempt from IRB requirements.

You agree to indemnify the Proprietor, and its licensees and assigns, against any claims that result from your breach of the above warranties.

Declaration of Conflicts of Interest.
You certify that:
1. All forms of financial support, including pharmaceutical company support, are acknowledged in the Contribution.
2. Any commercial or financial involvements that might present an appearance of a conflict of interest related to the Contribution are disclosed in the covering letter accompanying the Contribution and all such potential conflicts of interest will be discussed with the Editor as to whether disclosure of this information with the published Contribution is to be made in the Journal.
3. You have not signed an agreement with any sponsor of the research reported in the Contribution that prevents you from publishing both positive and negative results or that forbids you from publishing this research without the prior approval of the sponsor.
4. You have checked in the manuscript submission guidelines whether this Journal requires a Declaration of Conflicts of Interest and complied with the requirements specified where such a policy exists. It is not expected that the details of financial arrangements should be disclosed. If the Journal does require a Declaration of Conflicts of Interest and no conflicts of interest are declared, the following will be printed with your article: 'None Declared'.
5. You have checked the instructions to authors, and where declaration of grant funding is required, you have provided the appropriate information, in the format requested, within the submitted manuscript

Supplemental Material.
Supplemental Material includes all material related to the Article, but not considered part of the Article, provided to the Proprietor by you as the Contributor. Supplemental Material may include, but is not limited to, datasets, audio-visual interviews including podcasts (audio only) and vodcasts (audio and visual), appendices, and additional text, charts, figures, illustrations, photographs, computer graphics, and film footage. Your grant of a non-exclusive right and license for these materials to the Proprietor in no way restricts re-publication of Supplemental Material by you or anyone authorized by you.

Publishing Ethics & Legal Adherence.
Contributions found to be infringing this Agreement may be subject to withdrawal from publication (see Termination below) and/or be subject to corrective action. The Proprietor (and/or SAGE if SAGE is different than the Proprietor) reserves the right to take action including, but not limited to: publishing an erratum or corrigendum (correction); retracting the Contribution; taking up the matter with the head of department or dean of the author's institution and/or relevant academic bodies or societies; or taking appropriate legal action.

Contributor's Responsibilities with Respect to Third Party Materials.
You are responsible for: (i) including full attribution for any materials not original to the Contribution; (ii) securing and submitting with the Contribution written permissions for any third party materials allowing publication in all media and all languages throughout the world for the full legal term of copyright; and (iii) making any payments due for such permissions. SAGE is a signatory of the STM Permissions Guidelines, which may be reviewed online.

Termination.
The Proprietor, in its sole, absolute discretion, may determine that the Contribution should not be published in the Journal. If the decision is made not to publish the Contribution after accepting it for publication, then all rights in the Contribution granted to the Proprietor shall revert to you and this Agreement shall be of no further force and effect. Any payment made by you to the Proprietor will be refunded, and neither you nor the Proprietor will have any obligation to the other with respect to the Contribution. In the event that payment of the applicable APC is not received by the Proprietor, this Agreement will be terminated.

General Provisions.
The validity, interpretation, performance and enforcement of this Agreement shall be governed as follows: (1) where the Journal is published by SAGE's offices in the United Kingdom, by English law and subject to the jurisdiction and venue of the English courts; (2) where the Journal is published by SAGE's offices in the United States, by the laws of the State of California and subject to the jurisdiction and venue of the courts of the State of California located in Ventura County and of the U.S. District Court for the Central District of California; and (3) where the Journal is published by SAGE's offices in Southeast Asia, by the laws of India and subject to the jurisdiction and venue of the Indian courts.

In the event a dispute arises out of or relating to this Agreement, the parties agree to first make a good-faith effort to resolve such dispute themselves. Upon failing, the parties shall engage in non-binding mediation with a mediator to be mutually agreed on by the parties. Any controversy or claim arising out of or relating to this Agreement, or the breach thereof, which the parties cannot settle themselves or through mediation, shall be settled by arbitration.

This transaction may be conducted by electronic means and the parties authorize that their electronic signatures act as their legal signatures of this Agreement. This Agreement will be considered signed by a party when his/her/its electronic signature is transmitted. Such signature shall be treated in all respects as having the same effect as an original handwritten signature. (You are not required to conduct this transaction by electronic means or use an electronic signature, but if you do so, then you hereby give your authorization pursuant to this paragraph.)

This Agreement constitutes the entire agreement between the parties with respect to its subject matter, and supersedes all prior and contemporaneous agreements, understandings and representations. The full terms of the CC BY license may be accessed here: http://creativecommons.org/licenses/by/4.0/legalcode and the full terms of the CC BY-NC license may be accessed here: http://creativecommons.org/licenses/by-nc/4.0/legalcode. The terms of both licenses are incorporated herein by reference.

No amendment or modification of any provision of this Agreement shall be valid or binding unless made in writing and signed by all parties.

Consent for Commercial Electronic Messages.
You hereby provide your express consent for the Proprietor, its affiliates and licensees (expressly including SAGE, where SAGE is not the Proprietor), and their respective designees to contact you in connection with any business communication or other correspondence. The parties agree that such consent may be withdrawn by you at a later time by providing written notice (including by email) to the Proprietor (and/or SAGE if different than the Proprietor). This clause shall survive expiration or earlier termination of this Agreement.
